# (Fe_4_N/GaN)@GC: A Bifunctional Electrocatalyst for High‐Performance Rechargeable Zinc‐Air Batteries

**DOI:** 10.1002/advs.76564

**Published:** 2026-07-16

**Authors:** Xin‐Yuan Wei, Sai‐Sai Xie, Xia‐Li Ding, Yuan‐Qi Zhai, Sen Yu, Xiang‐Quan Hu, Rongqian Wu, Jintao Lu, Yan‐Zhen Zheng

**Affiliations:** ^1^ Frontier Institute of Science and Technology Interdisciplinary Research Center of Frontier Science and Technology State Key Laboratory of Electrical Insulation and Power Equipment Xi'an Key Laboratory of Electronic Devices and Materials Chemistry Xi'an Jiaotong University Xi'an P. R. China; ^2^ School of Future Technology Xi'an Jiaotong University Xi'an P. R. China; ^3^ Shaanxi Science and Technology Holding Institute Xi'an P. R. China; ^4^ Shaanxi Science and Technology Holdings Group Xi'an P. R. China; ^5^ Department of Hepatobiliary Surgery and Institute of Advanced Surgical Technology and Engineering The First Affiliated Hospital of Xi'an Jiaotong University Xi'an P. R. China; ^6^ Key Laboratory of the Ministry of Education and International Center for Dielectric Research School of Electronic Science and Engineering Xi'an Jiaotong University Xi'an P. R. China

**Keywords:** electrocatalyst, gallium nitride, heterostructure, iron nitride, zinc‐air battery

## Abstract

Rechargeable zinc‐air batteries (ZABs), though offering a high theoretical energy density (1086 Wh kg^−1^) and intrinsic nonflammability, require efficient electrocatalysts for oxygen reduction reaction (ORR) and oxygen evolution reaction (OER). At present, state‐of‐the‐art electrocatalysts for ORR and OER are dominated by noble metals, yet a single catalyst cannot deliver excellent activity for both reactions simultaneously due to distinct reaction kinetics. Herein, we report that a graphitized‐carbon‐encapsulated Fe_4_N/GaN heterostructure formulated as (Fe_4_N/GaN)@GC‐0.75 (FeGa/C‐0.75) delivers high‐performance ORR (half‐wave potential of 0.857 V) and OER (overpotential of 314 mV at 10 mA cm^−2^) electrocatalytic activities. Such a superior performance originates from the unprecedented Fe_4_N/GaN heterostructure, in which the adsorption of oxygenated intermediates can be optimized through interfacial electronic coupling of both nitrides. Meanwhile, the graphitic carbon shell protects the heterointerface against corrosion and decay. Therefore, ZABs assembled with FeGa/C‐0.75 show a high peak power density of 221 mW cm^−2^ with a specific capacity of 726.15 mAh g_Zn_^−1^ and maintain stable cycling for over 850 h at 30 mA cm^−2^. Moreover, a flexible all‐solid‐state ZAB enabled by FeGa/C‐0.75 can be fabricated with robust performance upon bending. These performances shed light on the broader application of this catalyst for various rechargeable ZABs and flexible devices.

## Graphical abstract

This work demonstrates that an unprecedented Fe_4_N/GaN heterostructure, stabilized by a graphitized carbon shell, induces interfacial charge redistribution and optimizes oxygen‐intermediate adsorption, enabling highly efficient bifunctional ORR/OER electrocatalysis for rechargeable zinc‐air batteries.

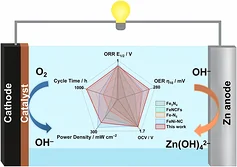

## 1 Introduction

Zinc‐air batteries (ZABs) offer a very high theoretical energy density (1086 Wh kg^−1^) and use nonflammable components, making them attractive for portable and flexible energy‐storage devices [1, 2, 3, 4]. However, the practical energy density of commercial ZABs remains far below the theoretical value, primarily due to the insufficient activity and durability of oxygen reduction reaction (ORR) and oxygen evolution reaction (OER) catalysts at the air cathode [5, 6, 7, 8]. Therefore, developing low‐cost, highly active, and durable bifunctional ORR/OER electrocatalysts is essential for the large‐scale application of rechargeable ZABs [9, 10].

Current commercial electrocatalysts for ORR and OER are primarily based on noble metals, such as Pt/C, IrO_2_, and RuO_2_. However, these catalysts are not only high‐cost but also insufficiently stable under alkaline conditions, which severely limits their practical application [6]. Moreover, each noble‐metal catalyst is only suitable to catalyze either ORR or OER, making a single catalyst insufficient to meet the bifunctional requirements of rechargeable ZABs [11, 12]. Accordingly, recent work has focused on developing non‐noble bifunctional ORR/OER catalysts with balanced activity and durability in alkaline media. However, many non‐noble systems suffer from a severe ORR‐OER activity trade‐off and inadequate stability under high‐current cycling. Fundamentally, this persistent trade‐off is dictated by the universal linear scaling relationships among oxygenated intermediates on a single geometric site, which impose a theoretical overpotential limit for bifunctional oxygen electrocatalysis [13, 14, 15, 16]. Such intrinsic thermodynamic constraints ultimately lead to large charge–discharge voltage gaps and rapid performance decay in practical devices [17, 18, 19].

Notably, transition‐metal nitrides provide promising performance owing to their noble‐metal‐like electronic structures [20, 21, 22, 23]. However, unmodified iron nitride phases (e.g., pure Fe_4_N) and conventional Fe‐N single‐atom catalysts often suffer from intrinsically sluggish OER kinetics under anodic conditions. More critically, the bifunctional activities of these conventional geometric sites are inherently restricted by rigid scaling relations among oxygen intermediates, which severely limit their high‐current durability [24, 25, 26, 27]. To mitigate these thermodynamic constraints, constructing heterostructures that create electronically differentiated adsorption environments provides a feasible strategy for balancing ORR and OER intermediate binding [28, 29, 30, 31]. Leveraging the capability of semiconducting GaN to modulate interfacial charge transfer, GaN is a suitable electronic‐structure modulator for heterostructure design [32, 33, 34]. As both are nitrides, GaN is intrinsically compatible with tetrairon nitride (Fe_4_N), which facilitates the formation of an intimate nitride‐nitride heterointerface [35, 36]. However, such a heterostructure has never been realized.

Conventional synthesis of metal nitrides typically requires high temperatures and reductive atmospheres (e.g., H_2_) to first convert metal oxides or salts into the metallic state, followed by long‐term nitridation in NH_3_, which not only leads to high energy consumption but also poses potential environmental risks associated with the use and emission of ammonia [37, 38, 39].

In this study, we develop a simple strategy without external NH_3_, in which melamine serves as a reductant as well as a carbon and nitrogen source under N_2_ atmosphere to rapidly construct a graphitized‐carbon‐encapsulated Fe_4_N/GaN heterostructure: (Fe_4_N/GaN)@GC‐0.75 (denoted as FeGa/C). The optimized FeGa/C‐0.75 (where 0.75 represents the n(Ga)/n(Fe) molar ratio) achieves an ORR half‐wave potential of 0.857 V and an OER overpotential of 314 mV at 10 mA cm^−2^. DFT calculations further indicate that GaN‐induced charge transfer optimizes the adsorption of oxygen intermediates, lowering the potential‐determining barrier for OER while moderating ORR intermediate binding, thereby rationalizing the bifunctional enhancement. With the protection from a graphitic carbon shell, the FeGa/C‐0.75 heterostructure delivers a peak power density of 221 mW cm^−2^ and cycles stably for over 850 h in ZABs. Comprehensive benchmarking indicates that this integrated Fe_4_N/GaN‐carbon architecture helps address the OER deficiency commonly encountered in traditional Fe‐N electrocatalysts, while retaining competitive ORR activity and long‐term device durability. Furthermore, its ultra‐long cycling stability (>850 h) positions this heterostructure among the top‐tier non‐noble electrocatalysts reported to date. Unlike conventional Fe‐based ORR catalysts or single‐component iron nitrides, this work focuses on the NH_3_‐free in situ construction of a carbon‐encapsulated Fe_4_N/GaN heterostructure that integrates interfacial electronic modulation, conductive carbon‐shell/CNT pathways, and structural protection for durable bifunctional oxygen electrocatalysis. Such performance has surpassed state‐of‐the‐art noble‐metal‐based catalysts and shed light on practical applications of this catalyst for rechargeable ZABs. We further demonstrate that an all‐solid‐state ZAB made from FeGa/C‐0.75 maintains an output of 20 mA cm^−2^ after bending and recovery for many cycles, indicating the potential as an energy source for flexible devices.

## 2 Results and Discussion

### 2.1 Structure and Composition Analysis

The synthesis process of FeGa/C is schematically illustrated in Figure 1a. The FeGa/C catalysts were prepared by urea‐assisted precursor formation followed by melamine‐assisted pyrolysis. During N_2_ pyrolysis, the urea‐derived Fe/Ga‐containing precursor and melamine‐derived decomposition products jointly create a local C/N‐rich and reducing/nitriding environment for the NH_3_‐free formation of Fe_4_N/GaN heterostructure nanoparticles [40, 41]. Concurrently, carbonization yields a carbon coating containing local graphitized domains [42, 43], and Fe catalyzes in situ CNT growth, producing a conductive CNT‐carbon network around the heterostructure [44, 45].

**FIGURE 1:**
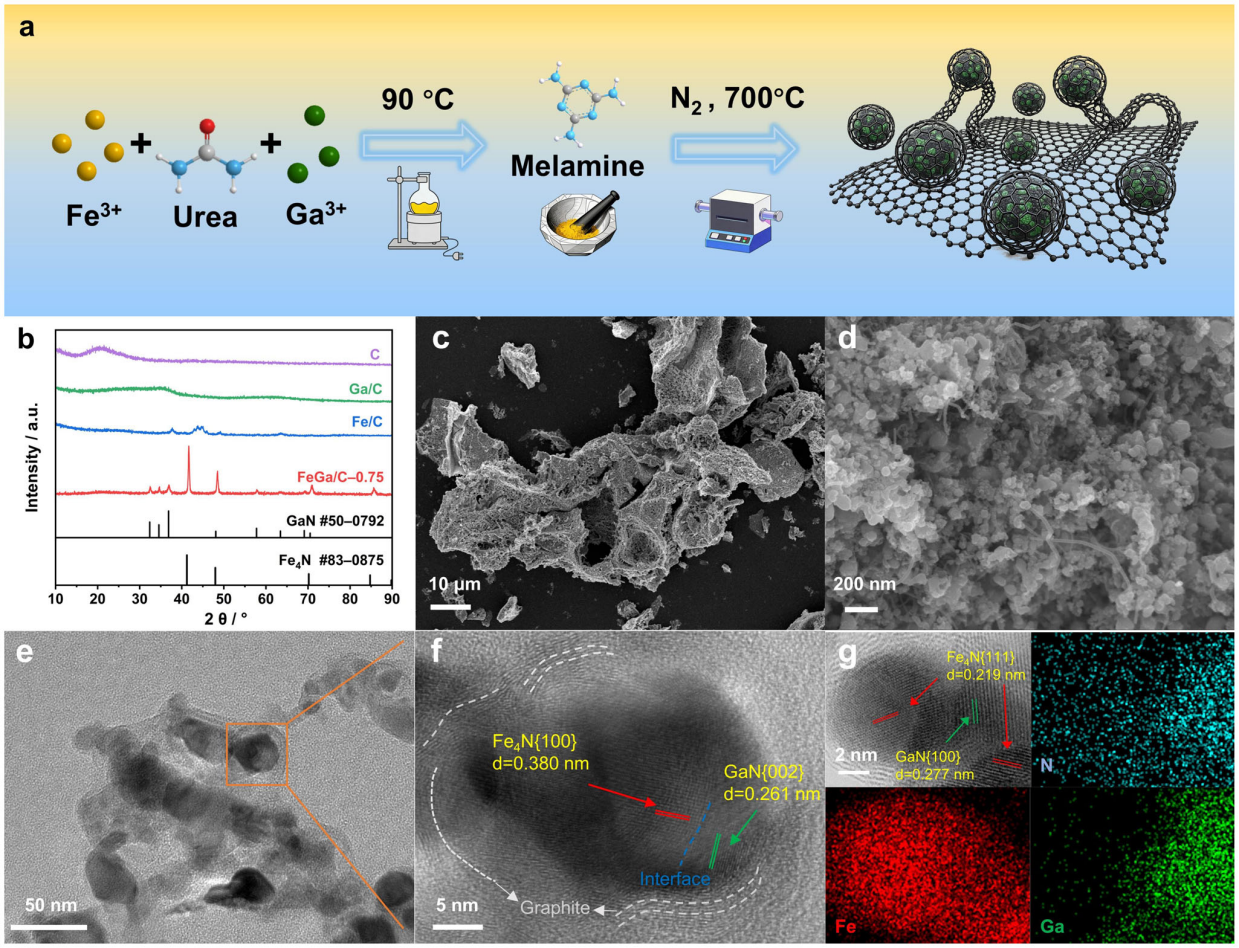
Synthesis and structural characterization of the Fe_4_N/GaN heterostructure encapsulated in graphitized carbon. (a) Schematic illustration of the synthesis of FeGa/C‐0.75. (b) XRD patterns of C, Ga/C, Fe/C, and FeGa/C‐0.75. (c,d) SEM images of FeGa/C‐0.75. (e,f) TEM images and g Aberration‐corrected STEM image and corresponding STEM‐EDS elemental maps of N, Fe, and Ga for FeGa/C‐0.75.

XRD (Figure 1b) indicates that FeGa/C‐0.75 contains both wurtzite GaN and cubic Fe_4_N phases. Characteristic reflections of GaN and Fe_4_N are observed, with partial overlap near 48° (Fe_4_N (200)/GaN (102)) and 70° (Fe_4_N (220)/GaN (201)), consistent with a heterostructured nitride composite. Control samples prepared under identical conditions (Ga/C and Fe/C) clarify phase evolution: Ga alone does not crystallize into GaN, whereas the presence of Fe promotes GaN formation; conversely, introducing Ga suppresses Fe_3_C formation and steers Fe toward nitridation, enabling Fe_4_N and GaN to form together. Across the FeGa/C‐x series (Figure S1), increasing the Ga precursor ratio strengthens the GaN‐related diffraction peaks, while the Fe_4_N phase is retained. A slight shift of the Fe_4_N diffraction peaks can be observed, which may originate from nanoscale interfacial strain or local lattice distortion induced by the neighboring GaN domains. To clarify whether the shift arises from homogeneous Ga doping or Fe_4_N/GaN interfacial coupling, aberration‐corrected STEM and local STEM‐EDS mapping were conducted (Figure 1g and Figure S22). The high‐resolution images show well‐resolved crystalline domains with lattice spacings assignable to Fe_4_N and GaN, respectively. The corresponding elemental maps reveal spatially adjacent Fe‐rich and Ga‐rich domains with N signals distributed across both regions, supporting a locally coupled Fe_4_N/GaN heterostructure. These observations do not support Ga‐doped Fe_4_N as the dominant structure, although minor interfacial atomic mixing cannot be completely excluded.

SEM/TEM images (Figure 1c–g and Figure S4) show that FeGa/C‐0.75 forms porous agglomerates composed of CNT‐interwoven carbon domains and 10–20 nm nitride nanoparticles. The nanoparticles are wrapped by thin carbon layers showing local graphitic lattice fringes, whereas the surrounding carbon matrix is mainly poorly ordered or amorphous. This structure indicates localized graphitization around the nitride nanoparticles, likely induced by Fe‐containing domains during pyrolysis. The resulting architecture can be described as carbon‐encapsulated Fe_4_N/GaN nanodomains embedded in a defect‐rich carbon/CNT framework, which provides electron‐transport pathways and structural protection. HRTEM (Figure 1f) shows two lattice‐fringe domains within a single nanoparticle covered by a thin carbon layer, confirming the coexistence of crystalline nitride domains within the carbon‐encapsulated structure. SAED further confirms the coexistence of cubic Fe_4_N and wurtzite GaN phases (Figure S2 and Table S1). Low‐magnification TEM‐EDS mapping further shows that Fe, Ga, and N are broadly co‐distributed over the catalyst aggregate, supporting the successful incorporation of Fe‐ and Ga‐containing nitride species at the mesoscale (Figure S21).

To further resolve the local Fe_4_N/GaN coupling, aberration‐corrected STEM characterization and corresponding STEM‐EDS elemental mapping were conducted (Figure 1g and Figure S22). The high‐resolution images reveal adjacent crystalline domains with lattice spacings assignable to Fe_4_N and GaN planes. In Figure S22, the lattice spacings of 0.218–0.219 nm and 0.242–0.243 nm correspond to Fe_4_N (111) and GaN (101), respectively. The corresponding elemental maps show spatially adjacent Fe‐ and Ga‐rich domains with N signals distributed across both regions, supporting the formation of a locally coupled Fe_4_N/GaN nitride heterostructure and excluding severe particle‐level elemental segregation. Because Fe salts can form Fe‐N_x_ coordination sites during pyrolysis with N‐containing carbon precursors, we further examined whether atomically dispersed Fe species dominate the catalyst surface. In the present synthesis, the relatively high Fe content and 700°C pyrolysis condition favor the formation of crystalline Fe‐containing nitride domains, as evidenced by XRD, SAED, HRTEM, and aberration‐corrected STEM/TEM analyses. Representative aberration‐corrected STEM/TEM images collected from carbon‐shell regions show crystalline Fe_4_N nanodomains wrapped by thin carbon layers, while no obvious isolated metal‐related atomic contrast is observed on the inspected carbon‐shell areas (Figure S22c,d). These observations indicate that the dominant Fe‐containing species are crystalline Fe_4_N nanodomains coupled with GaN, instead of abundant isolated Fe atoms decorating the carbon surface.

The protective role of the carbon shell against electrochemical corrosion was further examined by post‐OER structural characterization. Transition‐metal nitrides are generally susceptible to oxidation and structural degradation under anodic OER conditions. Therefore, post‐reaction HRTEM and elemental mapping analyses were conducted after OER operation (Figure S17). The HRTEM images show that the carbon‐encapsulated nanoparticle morphology is largely preserved, although slight local amorphization can be observed. The corresponding EDS elemental maps reveal that Fe and Ga remain spatially co‐localized in the observed post‐OER particles without obvious Fe/Ga spatial segregation. These results indicate that the Fe_4_N/GaN‐derived heterostructure is not catastrophically destroyed during OER operation. We note that partial surface oxygenation may still occur under high anodic potentials, as suggested by operando Raman spectroscopy, but the overall carbon‐encapsulated Fe/Ga‐containing framework remains structurally and compositionally stable under the tested conditions.

XPS was used to probe surface chemical states and electronic environments of C, Ga/C, Fe/C, and FeGa/C‐0.75 (C 1s at 284.8 eV for calibration). C 1s spectra (Figure 2a) are dominated by sp^2^ C─C/C═C with minor heteroatom‐containing species, indicating a graphitized carbon framework. Fe‐containing samples show an enhanced and narrowed sp^2^ peak, consistent with Fe‐assisted graphitization observed by Raman/TEM [46]. Raman spectra show D and G bands for all samples, indicating defect‐rich carbon frameworks containing locally graphitized domains. Fe‐containing samples exhibit slightly lower I*_D_*/I*_G_* ratios than Fe‐free samples, suggesting Fe‐assisted partial graphitization during pyrolysis (details in Figure S3).

**FIGURE 2:**
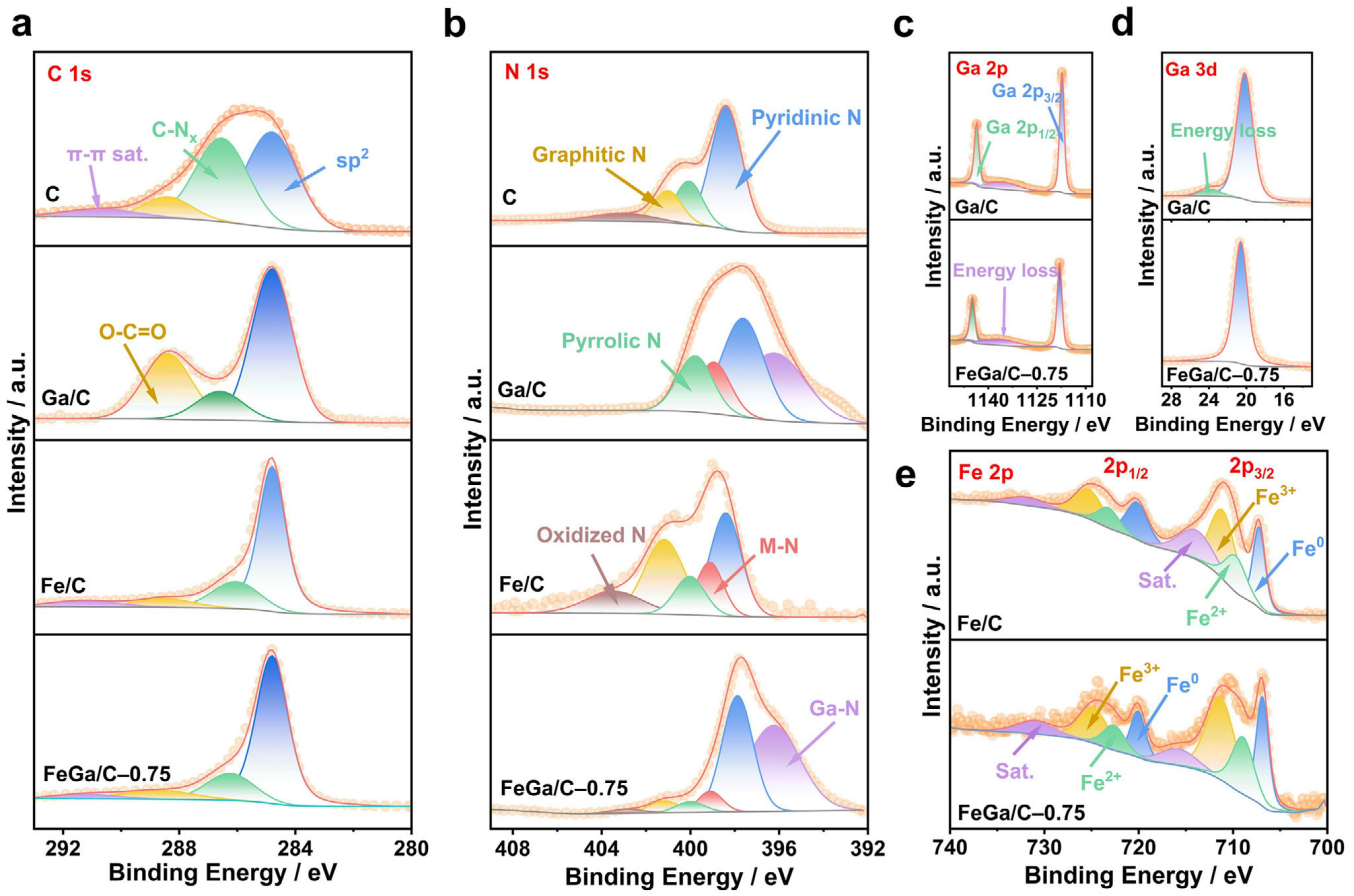
Surface chemical states and interfacial electronic modulation revealed by XPS analysis. (a) XPS C 1s spectra and (b) N 1s spectra of C, Ga/C, Fe/C, and FeGa/C‐0.75. (c) Ga 2p spectra of Ga/C and FeGa/C‐0.75. (d) Ga 3d spectra of Ga/C and FeGa/C‐0.75. (e) Fe 2p spectra of Fe/C and FeGa/C‐0.75.

N 1s spectra (Figure 2b) contain pyridinic, pyrrolic, graphitic, and oxidized N species. Metal‐containing samples show an additional M‐N component, and Ga‐containing samples exhibit a distinct Ga‐N signal at 396.1 eV, confirming the formation of nitride/coordination‐related N environments. Compared with Fe/C, FeGa/C‐0.75 shows suppressed oxidized‐N contributions and modified coordination/nitride‐related N features, consistent with Ga‐assisted interfacial electronic regulation. It should be noted that the M‐N component in the N 1s spectra should not be assigned to Fe‐N_4_ sites. In the present Fe_4_N/GaN/C system, this signal more likely contains contributions from nitride‐related Fe‐N/Ga‐N coordination environments and interfacial metal‐N bonding, together with possible minor Fe‐N_x_ species in the N‐doped carbon matrix. The apparent decrease in the surface N content from Ga/C to FeGa/C‐0.75 (39.73–22.17 at.%, Table S2) can be attributed to Fe‐directed restructuring of the melamine‐derived carbon/nitride matrix during pyrolysis. Fe promotes carbonization, partial graphitization, and in situ CNT growth, thereby decreasing labile N species such as pyrrolic and oxidized N while preserving nitride/coordination‐related N environments associated with Ga‐N and interfacial metal‐N bonding (Table S3). Therefore, the lower total surface N content does not directly indicate a reduced density of catalytically relevant interfacial sites. Instead, it reflects the transformation of an N‐rich but less conductive surface into a more conductive Fe_4_N/GaN‐carbon interfacial structure.

XPS reveals opposite core‐level shifts after Fe_4_N‐GaN coupling (Figure 2c–e): the Ga 3d/2p peaks shift to higher binding energies, while the Fe^0^/Fe^2+^ components shift to lower binding energies. Together with changes in N 1s features, these trends indicate interfacial electronic polarization and charge redistribution within the Ga‐N‐Fe environment, consistent with GaN‐induced tuning of oxygen‐intermediate adsorption. DFT calculations further support this interfacial polarization: the Fe d‐band center shifts toward the Fermi level (−2.31 to −2.20 eV), and Bader analysis indicates increased electron donation to the adsorbed oxygen intermediate after GaN modification. Together, XPS and DFT consistently indicate GaN‐driven electronic modulation that optimizes oxygen‐intermediate adsorption and promotes OER kinetics.

Because XPS probes only the near‐surface region and the nitride nanoparticles are wrapped by a carbon shell, ICP‐OES was further employed to determine the bulk Fe/Ga composition of the FeGa/C‐x series (Table S4). The measured Fe and Ga contents confirm that both elements are retained in the bulk catalysts. The Ga/Fe molar ratio increases from 0.20 to 0.77 with increasing Ga precursor feeding ratio, supporting the successful incorporation of Ga into the Fe‐containing catalyst series and confirming the designed composition trend.

### 2.2 Electrochemical Performance

ORR activity was evaluated in O_2_‐saturated 0.1 M KOH (three‐electrode setup; 1600 rpm for LSV; Figure 3a and Figure S6). FeGa/C‐0.75 delivers the most positive E_onset_ (0.98 V) and E_1/2_ (0.857 V) among the as‐prepared catalysts, comparable to commercial Pt/C (E_1/2_ = 0.85 V). It also shows a high diffusion‐limited current density (5.73 mA cm^−2^). Ga/C behaves similarly to the C sample, while Fe/C is intermediate, indicating that Ga incorporation alone is insufficient and that the in situ Fe_4_N/GaN heterostructure/interfacial coupling is key to boosting Fe‐based ORR sites. Tafel analysis in the kinetically controlled region (Figure 3b) shows FeGa/C‐0.75 has the smallest slope (62.3 mV dec^−1^), indicating faster ORR kinetics than Pt/C and the control catalysts.

**FIGURE 3:**
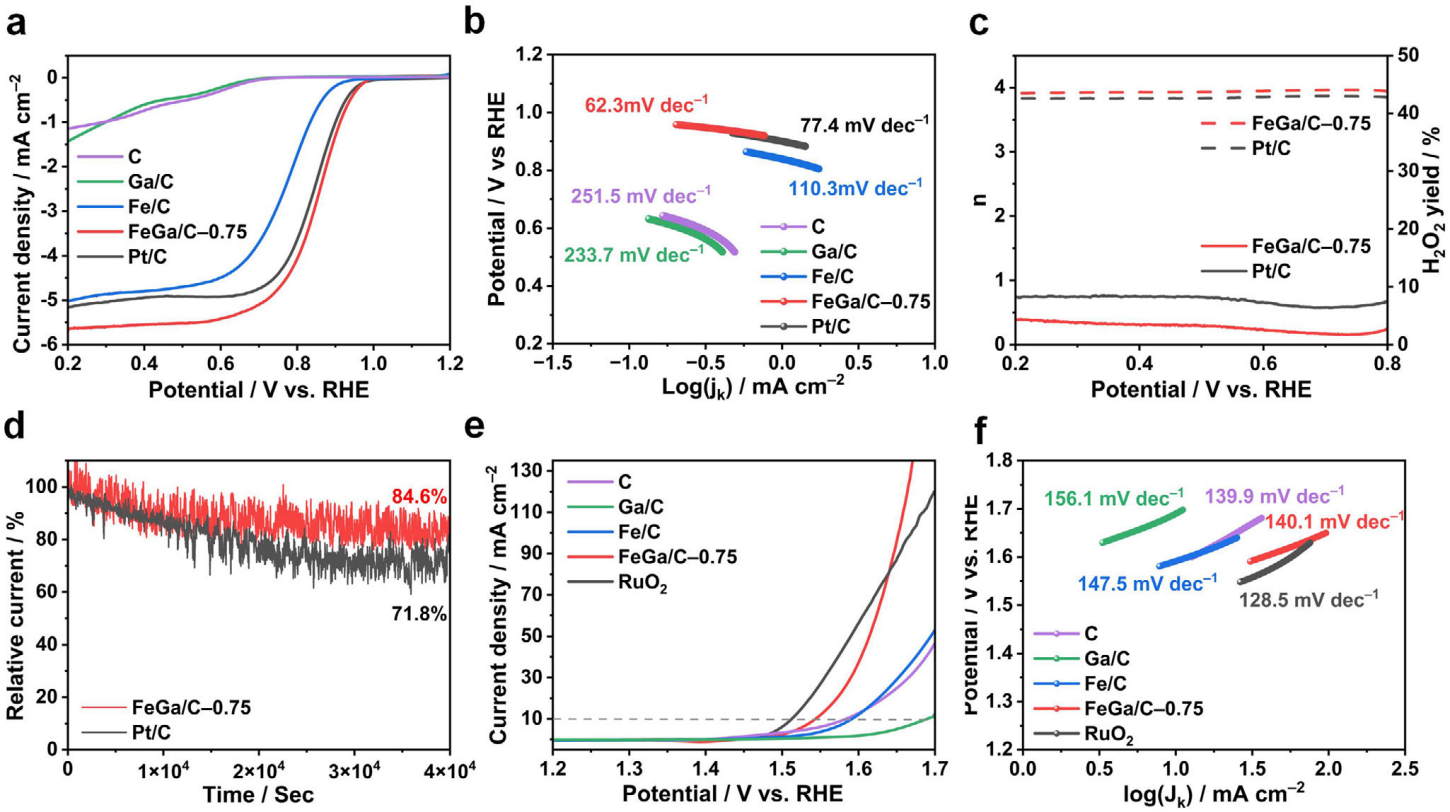
Bifunctional ORR and OER performance of FeGa/C‐0.75 in alkaline media. (a) ORR LSV curves of C, Ga/C, Fe/C, FeGa/C‐0.75, and Pt/C in O_2_‐saturated 0.1 M KOH (rotation rate: 1600 rpm; scan rate: 5 mV s^−1^). (b) Corresponding ORR Tafel plots of the catalysts. (c) H_2_O_2_ yield and electron‐transfer number (n) of FeGa/C‐0.75 and Pt/C obtained from RRDE measurements. (d) Chronoamperometric responses of FeGa/C‐0.75 and Pt/C at 0.6 V vs. RHE in O_2_‐saturated 0.1 M KOH (1600 rpm). (e) OER LSV curves after iR compensation of C, Ga/C, Fe/C, FeGa/C‐0.75, and RuO_2_ in 1.0 M KOH (rotation rate: 1600 rpm; scan rate: 5 mV s^−1^). (f) Corresponding OER Tafel plots of the catalysts.

To further investigate the ORR electron‐transfer kinetics of FeGa/C‐0.75, rotating disk electrode (RDE) polarization curves were recorded at different rotation rates (Figure S18). As the rotation speed increases, the current density increases accordingly, and a well‐defined diffusion‐limited current plateau appears in the high‐overpotential region. Based on Koutecký–Levich (K‐L) analysis at different potentials (inset), the K‐L plots of FeGa/C‐0.75 exhibit good linearity and nearly parallel slopes over a series of potentials, indicating that the ORR process is first order with respect to the dissolved oxygen concentration and proceeds with a relatively stable electron‐transfer number. From the slopes of the K‐L plots, the average electron‐transfer number of FeGa/C‐0.75 within the relevant potential window is 3.92, suggesting that ORR on FeGa/C‐0.75 is close to a four‐electron pathway [47].

Rotating ring‐disk electrode (RRDE) measurements further quantify the H_2_O_2_ yield and electron‐transfer number (Figure 3c) [48]. At 0.65 V, FeGa/C‐0.75 delivers n = 3.95 with an H_2_O_2_ yield as low as 2.2%, outperforming Pt/C (n = 3.86, H_2_O_2_ yield = 6.7%), indicating a near four‐electron pathway with suppressed peroxide formation compared with Pt/C.

ORR stability was evaluated by an accelerated potential‐cycling test (3000 cycles) and chronoamperometry (Figure S19 and Figure 3d). FeGa/C‐0.75 shows only a 22 mV loss in E_1/2_ after cycling and retains 84.6% of its current over 40 000 s, both better than Pt/C. The durability is attributed to the conductive CNT/graphitized‐carbon framework that protects and stabilizes the Fe_4_N/GaN active phase in alkaline media. ORR activity also depends on Ga content, reaching an optimum at x = 0.75 (Figure S7), consistent with interface‐enabled electronic‐structure optimization.

OER tests in 1.0 M KOH show that FeGa/C‐0.75 requires an overpotential of only 314 mV to reach 10 mA cm^−2^, comparable to RuO_2_ (295 mV) and better than Fe/C (360 mV), while Ga/C is nearly inactive (Figure 3e). The Tafel slope of FeGa/C‐0.75 is 140.1 mV dec^−1^, indicating improved OER kinetics (Figure 3f).

The OER performance of the FeGa/C‐x series (x = 0.25, 0.5, 0.75, and 1.0) shows a pronounced composition dependence (Figure S9). Increasing x from 0.25 to 0.75 substantially enhances the OER activity, indicating that an appropriate Ga content strengthens Fe4N/GaN interfacial modulation. Although FeGa/C‐1.0 exhibits comparable low‐current OER activity and a slightly lower Tafel slope, FeGa/C‐0.75 shows more favorable high‐current OER behavior and, more importantly, the best overall bifunctional balance when ORR activity, OER activity, and ZAB performance are considered together. This result suggests that a moderate Fe/Ga ratio is more beneficial for constructing an effective Fe_4_N/GaN‐carbon coupled architecture.

To clarify whether the enhanced activity originates from increased accessible surface area or improved intrinsic activity, the ECSA values were estimated from the double‐layer capacitance (C_dl_) using ECSA = C_dl_/C_s_, where C_s_ = 0.04 mF cm^−2^. The calculated ECSA values of Fe/C, FeGa/C‐0.25, FeGa/C‐0.5, FeGa/C‐0.75, and FeGa/C‐1 are 74.99, 41.15, 61.63, 46.82, and 42.78, respectively (Table S5). Notably, FeGa/C‐0.75 does not possess the largest ECSA, indicating that its superior ORR/OER activity cannot be assigned to an enlarged electrochemically accessible surface area alone. Furthermore, ECSA‐normalized ORR and OER polarization curves were added to compare the surface‐area‐corrected activity of the FeGa/C‐x series (Figure S14f,g). After normalization by the C_dl_‐derived ECSA, FeGa/C‐0.75 still shows superior ORR activity in the kinetically controlled region and strong OER activity, particularly at higher current densities. These results confirm that the enhanced bifunctional performance is mainly associated with improved intrinsic activity enabled by Fe_4_N/GaN interfacial electronic modulation.

Notably, although the Fe_4_N main phase remains essentially unchanged across FeGa/C‐x, the ORR and OER activities exhibit a pronounced x‐dependence (Figures S7 and S9), indicating that the enhancement cannot be explained solely by the Fe phase but is closely related to Ga‐induced interfacial/electronic modulation.

EIS measurements (Figure S11) indicate that C and Ga/C suffer from sluggish interfacial charge transfer, whereas Fe‐containing catalysts exhibit markedly reduced charge‐transfer resistance. Despite a slightly higher resistance than Fe/C, FeGa/C‐0.75 maintains efficient charge transfer. Together with the ORR/OER results, these observations suggest that the superior bifunctional activity of FeGa/C‐0.75 originates from the synergistic charge‐transport characteristics and interfacial electronic modulation.

Operando Raman spectroscopy was further conducted to probe the working‐state evolution of FeGa/C‐0.75 under ORR‐ and OER‐relevant potentials (Figure S20). During ORR polarization, several reversible low‐wavenumber features appear in the 400–800 cm^−1^ region, which can be associated with oxygenated adsorbate‐related vibrations, such as M‐O(H)‐related species involved in oxygen adsorption and reduction. Under OER polarization, only weak and broad M‐O(H)‐related/oxygenated‐species signals are detected at high anodic potentials, especially near 1.6–1.7 V vs. RHE, suggesting partial surface oxygenation or adsorbed oxygenated species during OER operation. Meanwhile, the carbon D and G bands remain detectable under both ORR and OER polarization, indicating that the carbon framework is spectroscopically retained during electrochemical operation. Combined with the post‐OER HRTEM/EDS results, the operando Raman spectra suggest that FeGa/C‐0.75 may undergo limited surface oxygenation under high anodic potentials, while the overall carbon‐encapsulated Fe/Ga‐containing framework remains preserved. Therefore, the enhanced OER durability should be attributed to the protective carbon shell and the stable Fe_4_N/GaN‐derived interfacial structure.

To evaluate the intrinsic ORR/OER activity of Fe_4_N without GaN modification, a GaN‐free Fe_4_N control was prepared. Following a reported procedure, hexamethylenetetramine (HMTA) was added during synthesis to promote nitridation, yielding a carbon‐supported, phase‐pure Fe_4_N sample, denoted as Fe/C‐HMTA. The phase purity was confirmed by XRD (Figure S15). For comparison, Fe/C‐HMTA was mechanically mixed with commercial GaN at a mass ratio of 1:1 to obtain Fe/C‐HMTA+GaN, which represents a non‐in situ contact between the two components. As shown in Figure S16, Fe/C‐HMTA and Fe/C‐HMTA+GaN exhibit comparable ORR and OER performances. By contrast, the in situ synthesized FeGa/C‐0.75 shows much higher bifunctional activity. Together with the C, Ga/C, and Fe/C controls, these comparisons indicate that the enhanced activity cannot be attributed to isolated carbon, Ga‐containing species, Fe‐containing phases, or physically mixed GaN. The activity enhancement of FeGa/C‐0.75 is therefore mainly associated with the intimate Fe_4_N/GaN‐carbon coupling formed during pyrolysis.

### 2.3 DFT Calculations to Elucidate ORR/OER Mechanisms

To gain insight into how GaN modulates Fe_4_N, first‐principles calculations based on DFT were performed using the *Vienna Ab initio Simulation Package* (VASP) [49]. Figure 4a shows the Fe_4_N/GaN/C heterostructure model and representative adsorption configurations (^*^O, ^*^OH, and ^*^OOH) used for the ORR/OER analyses. The DFT model was constructed as an idealized Fe4N/GaN/C interfacial motif to evaluate how GaN introduction affects the electronic structure and oxygen‐intermediate adsorption energetics. The calculations suggest that oxygen intermediates exhibit different energetically preferred adsorption motifs in the idealized Fe_4_N/GaN/C model. ORR intermediates are favorably stabilized at Fe‐related interfacial motifs, whereas OER intermediates are stabilized on electronically perturbed carbon‐shell/interfacial C motifs. These assignments should be regarded as thermodynamic model indications, not definitive operando identification of exclusive catalytic centers.

**FIGURE 4:**
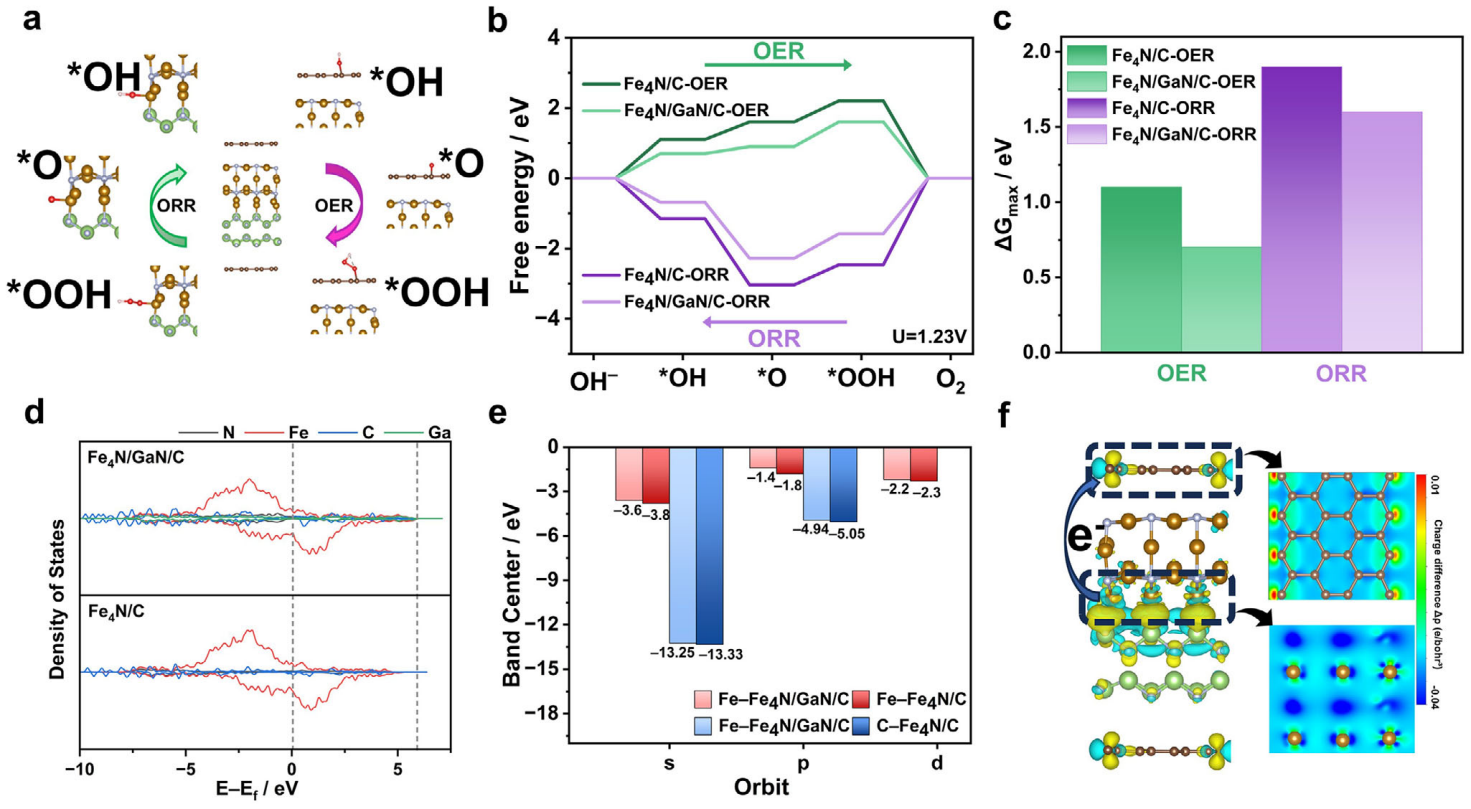
Theoretical insights into GaN‐modulated ORR/OER energetics and interfacial charge transfer. (a) The Fe_4_N/GaN/C composite model used in the electrocatalytic calculations and the optimized adsorption configurations of oxygen intermediates (^*^O, ^*^OH, and ^*^OOH). (b) Reaction free‐energy evolution of the studied systems along the OER and ORR pathways at U = 1.23 V vs. RHE before and after GaN modification. (c) Maximum uphill free‐energy change (ΔG_max_) extracted from the free‐energy diagrams for OER/ORR. (d) Density of states of each element before and after GaN modification. (e) Quantitative analysis of the observed energy‐level shifts via the changes in the s‐, p‐, and d‐band centers. (f) Differential charge‐density distribution at the Fe_4_N/GaN/C composite interface.

At U = 1.23 V vs. RHE, the free‐energy profiles of OER and ORR on Fe_4_N with and without GaN modification are summarized in Figure 4b [50]. Along the OER pathway (^*^OH → ^*^O → ^*^OOH → O_2_), GaN modification reshapes intermediate energetics, lowering the barrier associated with the potential‐determining step. For ORR, Fe‐based sites often show overly strong binding of ^*^O and ^*^OH, which can impede subsequent reduction steps [51]. After GaN modification, the energies of ^*^OOH, ^*^O, and ^*^OH shift upward, and the free‐energy profile becomes less steep, suggesting moderated intermediate binding.

Figure 4c compares the maximum uphill free‐energy change (ΔG_max_, the potential‐determining step) for OER and ORR. Introducing GaN decreases ΔG_max_ for OER from 1.1 to 0.7 eV, indicating an improved OER thermodynamic landscape in the model. Simultaneously, ΔG_max_ for ORR decreases from 1.90 to 1.60 eV. These ΔG_max_ values are used for model‐to‐model comparison of adsorption thermodynamics and should not be directly equated with the experimentally measured overpotential.

To see the electronic origin of these trends, we analyzed the spin‐polarized density of states (DOS) and interfacial charge redistribution (Figure 4d). The GaN‐modified structure exhibits a higher DOS near the Fermi level (EF), which is consistent with enhanced electronic states available around EF. In addition, the XPS results suggest electron redistribution between Ga species and Fe species, which shows interfacial charge transfer. Correspondingly, the Fe 3d‐band center shifts slightly toward EF (from −2.31 to −2.20 eV, Figure 4e), implying a modified electronic structure of Fe after GaN introduction. Such electronic modulation can affect the interaction between the carbon shell and oxygenated adsorbates through interfacial coupling, thereby tuning the adsorption energetics of OER intermediates.

Differential charge‐density analysis (Figure 4f) visualizes GaN‐induced charge polarization at the interface. Charge depletion is mainly observed in the GaN region, whereas charge accumulation extends toward the Fe_4_N domain and the surface carbon/adsorbate region. Bader charge analysis shows an increased net charge transfer to the adsorbate by 0.35 e after GaN modification, supporting enhanced electron donation to oxygenated intermediates. Together, the DOS, d‐band shift, and charge‐density analyses suggest that GaN‐induced interfacial charge redistribution can tune the binding strength of oxygen intermediates on the carbon surface, which is consistent with the reduced ΔG_max_ and the improved OER thermodynamics.

Crucially, these computational results suggest a spatially decoupled bifunctional mechanism within the (Fe_4_N/GaN)@GC architecture, which successfully alleviates the scaling‐imposed ORR/OER trade‐off. A central challenge in developing bifunctional electrocatalysts is intrinsic scaling relationships, where a single active site often cannot simultaneously optimize the adsorption energetics required for both ORR and OER intermediates. In the present system, the Fe_4_N/GaN heterostructure appears to alleviate this trade‐off by differentiating the energetically favorable regions for the two reactions. Specifically, the thermodynamic analysis suggests that OER is preferentially promoted on the outer graphitized carbon shell electronically modulated by the underlying Fe_4_N/GaN heterointerface, whereas ORR is more favorable at the interfacial Fe─N/Ga─N coordination environments.

It should be noted that the Fe_4_N/GaN/C model used here is an idealized comparative model. Its main purpose is to evaluate how GaN introduction modifies the electronic structure of the Fe_4_N/GaN/C architecture and influences the adsorption energetics of oxygen intermediates. The model does not aim to fully reproduce the dynamically evolved surface under high anodic OER potentials. Consistently, operando Raman spectra show only weak M‐O(H)‐related/oxygenated‐species signals at high OER potentials, suggesting partial surface oxygenation during operation. Therefore, the calculated OER energetics should be interpreted as evidence for Fe_4_N/GaN‐induced interfacial electronic modulation of oxygen‐intermediate adsorption, not as a definitive atomic‐level description of a fully reconstructed OER working surface.

Importantly, although the OER‐related adsorption sites in the model are located on the carbon shell, their electronic properties are influenced by the underlying Fe_4_N/GaN heterostructure. The GaN‐induced interfacial charge redistribution, evidenced by DOS modulation, Fe d‐band center upshift, and differential charge‐density analysis, electronically perturbs the adjacent carbon surface and tunes its interaction with oxygenated intermediates. Such interfacial electronic coupling lowers the potential‐determining barrier for OER and moderates the adsorption strength of ORR intermediates at Fe‐related interfacial sites. We emphasize that these site assignments should be regarded as mechanistic indications derived from thermodynamic models, not as definitive operando identification of the dominant catalytic centers. Under practical anodic conditions, partially oxygenated surface species may form, as suggested by operando Raman results. Overall, GaN‐induced Fe_4_N/GaN interfacial coupling optimizes oxygen‐intermediate adsorption at Fe‐related interfacial motifs for ORR and electronically regulated carbon‐shell/interfacial C sites for OER, while the local graphitized carbon shell/CNT framework also facilitates charge transport and structural stability, jointly accounting for the enhanced bifunctional activity of FeGa/C‐0.75.

### 2.4 Zinc‐Air Battery Performance

To evaluate device‐level performance, rechargeable aqueous ZABs were assembled using FeGa/C‐0.75 as the air cathode catalyst, with a mixed Pt/C + RuO_2_ catalyst (mass ratio 1:1) employed as a benchmark. A schematic illustration of the ZAB configuration is shown in Figure 5a. During discharge, the catalyst layer on the air cathode promotes ORR, in which O_2_ from air gains electrons to form OH^−^. Simultaneously, Zn at the anode is oxidized, releasing electrons to the external circuit and reacting with the OH^−^ rich alkaline electrolyte to form zinc hydroxide species. The continuous electron flow through the external circuit delivers electrical energy. Upon charging, the electrochemical processes proceed in the reverse direction: Zn species are reduced and redeposited as metallic Zn at the anode, while the air cathode switches to catalyze OER, regenerating O2 and completing the reversible zinc‐air chemistry.

**FIGURE 5:**
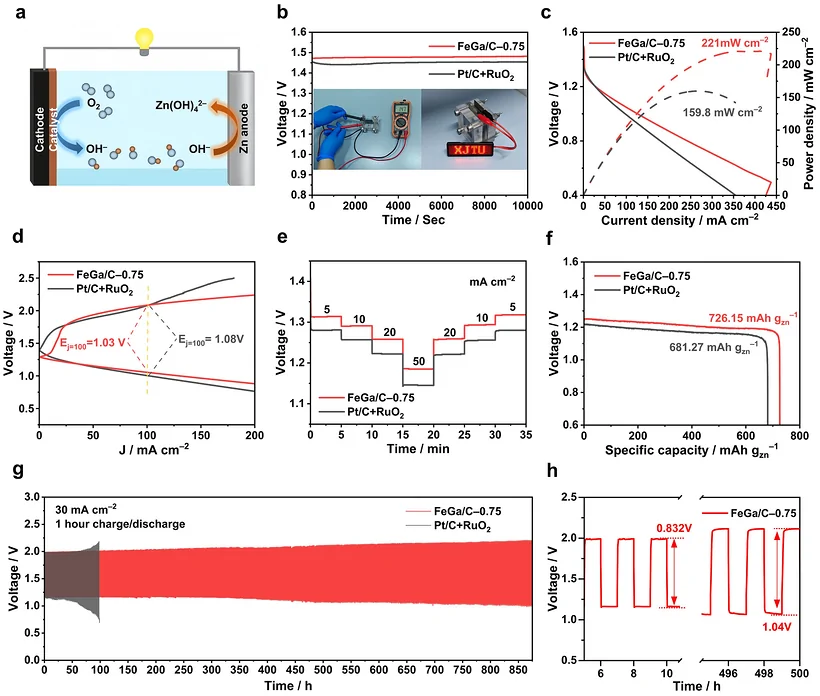
Electrochemical performance of aqueous rechargeable zinc‐air batteries based on FeGa/C‐0.75. (a) Schematic diagram of the aqueous rechargeable ZAB. (b) Open‐circuit voltage of ZABs using FeGa/C‐0.75 or Pt/C + RuO_2_ as the air cathode (inset: photographsKoutecký of a ZAB equipped with FeGa/C‐0.75 catalyst showing an open‐circuit voltage of 1.47 V, and a red LED powered by a single FeGa/C‐0.75‐based ZAB). (c) Discharge polarization curves and the corresponding power density plots of ZABs with FeGa/C‐0.75 and Pt/C + RuO_2_ air cathodes. (d) Galvanostatic charge–discharge profiles of ZABs with FeGa/C‐0.75 and Pt/C + RuO_2_ air cathodes. (e) Galvanostatic discharge curves of the FeGa/C‐0.75‐based ZAB at different current densities. (f) Specific capacities of Pt/C + RuO_2_‐ and FeGa/C‐0.75‐based ZABs at 20 mA cm^−2^ (normalized to the consumed Zn mass). (g,h) Long‐term galvanostatic charge–discharge cycling performance of Pt/C + RuO_2_ and FeGa/C‐0.75‐based ZABs at 30 mA cm^−2^.

Figure 5b presents the open‐circuit voltage of the ZABs, and the insets show photographs of the assembled FeGa/C‐0.75‐based device and a red LED powered by a single ZAB. Under open‐circuit conditions, the ZAB using FeGa/C‐0.75 as the air cathode exhibits an open‐circuit voltage (OCV) of approximately 1.47 V, which is slightly higher than that of the Pt/C + RuO_2_‐based ZAB (1.45 V). As revealed by the power density curves (Figure 5c), the FeGa/C‐0.75‐based ZAB delivers a peak power density of 221 mW cm^−2^, significantly higher than that of the Pt/C + RuO_2_‐based ZAB (159.8 mW cm^−2^). The charge–discharge polarization curves (Figure 5d) show that, at the same current density, the charge–discharge voltage gap of the FeGa/C‐0.75‐based ZAB is about 1.03 V, smaller than that of the Pt/C + RuO_2_‐based ZAB (1.08 V). These results indicate that FeGa/C‐0.75 exhibits superior bifunctional catalytic performance and energy efficiency at the device level.

The FeGa/C‐0.75‐based ZAB maintains stable discharge plateaus from 5 to 50 mA cm^−2^ and shows nearly full voltage recovery when switching back to lower currents, demonstrating good rate capability (Figure 5e). At 20 mA cm^−2^, it delivers a specific capacity of 726.15 mAh g_Zn_^−1^ (88.6% of Zn's theoretical capacity), higher than the combination of Pt/C + RuO_2_ catalysts (681.27 mAh g_Zn_^−1^; Figure 5f). Long‐term cycling at 30 mA cm^−2^ remains stable for over 850 h with only slow voltage decay, whereas the Pt/C + RuO_2_ cell operates for less than 100 h under the same protocol (Figure 5g,h). The excellent cycling durability is consistent with the preserved Fe_4_N/GaN heterostructure protected by the graphitized carbon shell after prolonged OER operation.

To quantitatively evaluate the practical merit of FeGa/C‐0.75, its catalytic activity and ZAB performance were compared with recently reported iron nitride and Fe‐N single‐atom electrocatalysts in alkaline media (Table 1 and Figure 6). The comparison includes ORR half‐wave potential, OER overpotential at 10 mA cm^−2^, open‐circuit voltage, peak power density, and cycling stability of rechargeable ZABs. Several representative Fe‐N_4_ and iron nitride catalysts exhibit excellent ORR activity; however, their OER activity or long‐term rechargeable ZAB cycling stability is often absent or less competitive. FeGa/C‐0.75 shows an ORR half‐wave potential of 0.857 V and a low OER overpotential of 314 mV, demonstrating balanced bifunctional oxygen electrocatalysis. At the device level, the FeGa/C‐0.75‐based ZAB delivers a peak power density of 221 mW cm^−2^ and maintains stable cycling for over 850 h at 30 mA cm^−2^. Compared with representative Fe‐based oxygen electrocatalysts, including iron nitrides and Fe‐N‐C/single‐atom catalysts, FeGa/C‐0.75 delivers balanced ORR/OER activity and robust rechargeable ZAB durability, highlighting the merit of the in situ‐constructed Fe_4_N/GaN‐carbon architecture.

**TABLE 1:** Quantitative comparison of alkaline ORR/OER activity and rechargeable ZAB performance of FeGa/C‐0.75 with recently reported iron nitride and Fe‐N single‐atom electrocatalysts.

Catalyst	ORR E_1/2_ / V	OER η_10_ / mV	Open Circuit Voltage / V	Power Density / mW cm^−2^	Cycle Time / h
This work	0.857	314	1.47	221	850
FeNCFs [52]	0.84	400	1.48	173	165
Fe‐N_4_ [53]	0.83	455	1.43	71.6	30
Fe‐N_5_ [54]	0.9	360	1.52	107.12	400
FeNi‐NC [55]	0.9	351	1.44	266.4	500
Cl‐modulated Fe‐N‐C [56]	0.91	/	1.51	183.5	150
Y_2_O_3_ boosted Fe‐N_4_ and Fe_4_N [57]	0.926	/	1.483	233	/
Fe_4_N embedded in nitrogen‐doped carbon [58]	0.904	/	1.49	119.6	180
Fe_2_N with a single Fe‐atom [59]	0.957	/	1.47	202	/

**FIGURE 6:**
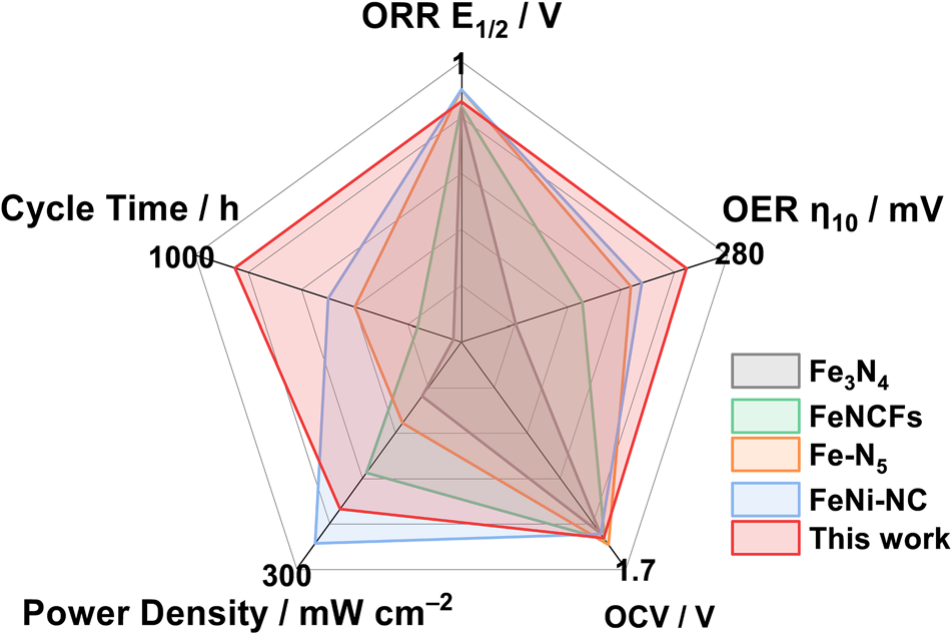
Multidimensional radar chart comparing the ORR half‐wave potential E_1/2_, OER overpotential at 10 mA cm^−2^, open‐circuit voltage, power density, and cycling stability of FeGa/C‐0.75 with recently reported iron nitride and Fe‐N single‐atom catalysts.

To demonstrate applicability in flexible/wearable electronics, a gel‐electrolyte‐based flexible ZAB was assembled using FeGa/C‐0.75, Zn foil, and a PAA‐KOH gel electrolyte (Figure 7a). The device can power a red LED (Figure 7b) and deliver peak power densities of 85.6 and 76.6 mW cm^−2^ in the flat and 90° bend states, respectively (Figure 7c). EIS and cycling results show similar impedance and stable operation in flat and bent states (Figure 7d,e). The intertwined graphitized carbon/CNT framework provides fast electron pathways and forms a mechanically resilient skeleton. Together with the flexible carbon paper substrate and gel electrolyte, the device maintains a stable output under bending. Consistently, the output remains stable during repeated bending/twisting when attached to the forearm (Movies S1 and S2), supporting the mechanical reliability of the flexible cell.

**FIGURE 7:**
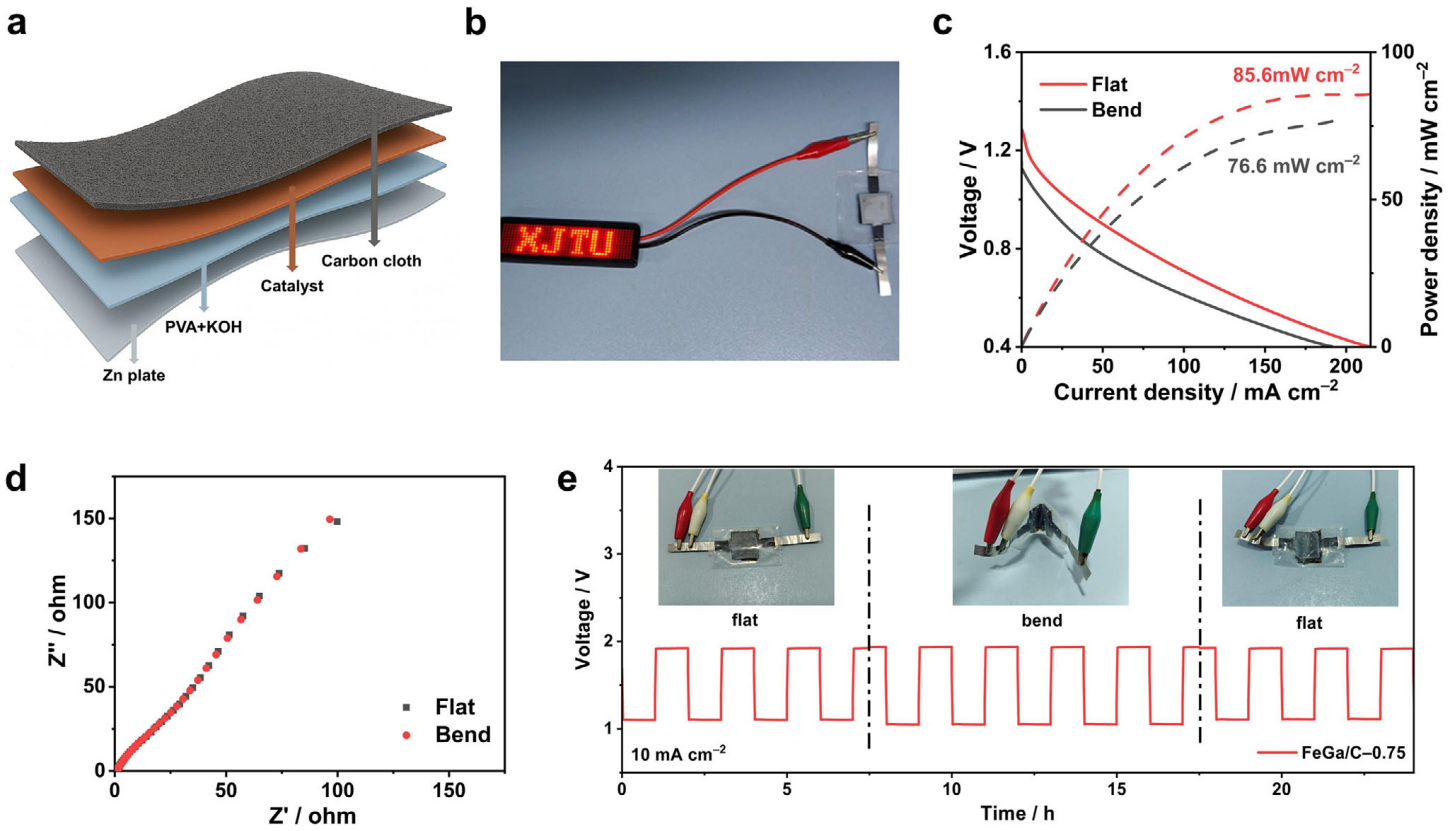
Performance of flexible all‐solid‐state zinc‐air batteries enabled by FeGa/C‐0.75. (a) Schematic diagram of the flexible all‐solid‐state ZAB. (b) Photograph of a red LED powered by a flexible ZAB using FeGa/C‐0.75 as the air cathode. (c) Discharge polarization curves and the corresponding power density plots of the FeGa/C‐0.75‐based flexible ZAB in flat and 90° bent states. (d) Nyquist plots of the FeGa/C‐0.75‐based flexible ZAB measured at the open‐circuit voltage (OCV) in the flat and 90° bend states. (e) Cycling performance of the FeGa/C‐0.75‐based flexible ZAB at 10 mA cm^−2^ in the flat state and after bending and recovery. The flexible ZAB was manually bent to approximately 90°, corresponding to an estimated inner bending radius of 5 mm.

## 3 Conclusions

In summary, we present a facile and eco‐friendly, NH_3_‐free strategy to construct a graphitized‐carbon‐encapsulated Fe_4_N/GaN heterostructure bifunctional electrocatalyst (FeGa/C). Spectroscopic evidence and DFT reveal strong Fe_4_N‐GaN interfacial coupling that drives charge polarization and tunes oxygen‐intermediate adsorption for both ORR and OER. The graphitized carbon shell, together with a CNT network, ensures fast electron transport and helps preserve the carbon‐encapsulated Fe/Ga‐containing framework during prolonged anodic operation in alkaline electrolyte.

The optimized FeGa/C‐0.75 achieves a competitive performance (ORR half‐wave potential of 0.857 V and an OER overpotential of 314 mV at 10 mA cm^−2^) compared with noble‐metal‐based catalysts. Unlike conventional Fe‐based systems and Fe‐N single‐atom electrocatalysts, this heterostructure mitigates the ORR/OER activity trade‐off by creating electronically differentiated adsorption environments for oxygen intermediates, while the carbon shell/CNT framework contributes to charge transport and structural stability against oxidative degradation. In practical ZABs, FeGa/C‐0.75 delivers a peak power density of 221 mW cm^−2^ and maintains stable cycling for over 850 h, far surpassing the combination of Pt/C + RuO_2_ catalysts (stable cycling for less than 100 h) under identical conditions, representing a notable advance in device‐level durability. The practicality is further validated in flexible all‐solid‐state ZABs, which retain robust performance under repeated mechanical deformation. Thus, this work provides a new design for a low‐cost, high‐performance bifunctional catalyst that can be used in rechargeable ZABs.

## 4 Experimental Section/Methods

### 4.1 Synthesis of the FeGa/C‐0.75 Catalyst

In a typical synthesis, Fe(NO_3_)_3_·9H_2_O (2.0 mmol) and Ga(NO_3_)_3_·9H_2_O (1.5 mmol) were first dissolved in 300 mL of deionized water, followed by the addition of urea (0.1 mol). The resulting solution was heated to 90°C and maintained at this temperature for 4 h under continuous magnetic stirring during the process. During the reaction, a yellow precipitate gradually formed due to the thermal decomposition of urea. After completion, the suspension was naturally cooled to room temperature, and the precipitate was collected by centrifugation, thoroughly washed with deionized water several times, and then dried at 60°C to obtain a yellow powder precursor.

The dried precursor was subsequently mixed with melamine (50 mmol) in an agate mortar. A small amount of anhydrous ethanol (1–2 mL) was added to aid homogeneous mixing. The mixture was ground thoroughly to form a uniform slurry, followed by drying to remove ethanol. The dried solid was further ground to obtain a homogeneous precursor powder.

The precursor powder was transferred to a tube furnace and calcined at 700°C with a heating rate of 5°C min^−1^ under a flowing N_2_ atmosphere (100 mL min^−1^), and maintained at this temperature for 2 h. After naturally cooling to room temperature under N_2_, the obtained product, denoted as FeGa/C‐0.75, was collected for further characterization.

### 4.2 Synthesis of Control Catalysts

To investigate the effect of the Fe/Ga molar ratio on the structure and electrochemical performance, a series of samples was prepared by varying the feeding molar ratio of Ga(NO_3_)_3_·9H_2_O to Fe(NO_3_)_3_·9H_2_O. The resulting catalysts were denoted as FeGa/C‐0.25, FeGa/C‐0.5, FeGa/C‐0.75, and FeGa/C‐1.0 (where the number x represents the n(Ga)/n(Fe) molar ratio).

For comparison, C, Fe/C, and Ga/C were prepared by the same procedure. In the case of C, no metal nitrates were added; for Fe/C, Ga(NO_3_)_3_·9H_2_O was omitted; and for Ga/C, Fe(NO_3_)_3_·9H_2_O was omitted.

It should be noted that in this synthesis, Ga and Fe precursors mutually facilitate the formation of Fe_4_N and GaN, respectively. Therefore, the Ga/C and Fe/C samples are used as reference materials prepared under identical conditions rather than as phase‐pure GaN or Fe_4_N controls.

Fe/C‐HMTA was synthesized following the same procedure as Fe/C, except that half of the melamine was replaced by hexamethylenetetramine (HMTA) [36]. Briefly, Fe(NO_3_)_3_·9H_2_O (2.0 mmol) was dissolved in 300 mL of deionized water, followed by the addition of urea (0.1 mol). The solution was heated to 90°C and maintained for 4 h under magnetic stirring. After naturally cooling to room temperature, the precipitate was collected by centrifugation, thoroughly washed with deionized water several times, and dried at 60°C to obtain the precursor powder.

The dried precursor was then mixed with melamine (25 mmol, 3.15 g) and HMTA (25 mmol, 3.50 g) in an agate mortar. A small amount of anhydrous ethanol (1–2 mL) was added to assist homogeneous mixing. The mixture was ground thoroughly to form a uniform slurry, followed by drying to remove ethanol. The dried solid was further ground to obtain a homogeneous powder, which was subsequently calcined at 700°C (heating rate: 5°C min^−1^) under flowing N_2_ (100 mL min^−1^) for 2 h. After naturally cooling to room temperature under N_2_, the obtained product was collected and denoted as Fe/C‐HMTA.

Fe/C‐HMTA+GaN was prepared by mechanically mixing Fe/C‐HMTA with commercial GaN at a mass ratio of 1:1. Typically, equal masses of Fe/C‐HMTA and GaN were dispersed in a small amount of anhydrous ethanol, followed by vigorous grinding in an agate mortar for 20–30 min to ensure homogeneous mixing. The resulting slurry was dried at 60°C to remove ethanol and then lightly ground again to obtain a uniform powder, which was denoted as Fe/C‐HMTA+GaN.

### 4.3 Electrocatalytic Measurements

The electrocatalytic performance of the catalysts was evaluated on a CHI 760E electrochemical workstation (CHI, Shanghai, Inc.) using a rotating disk electrode (RDE) technique in a conventional three‐electrode configuration. A Hg/HgO electrode and a graphite rod were employed as the reference and counter electrodes, respectively, while a glassy carbon RDE with a geometric area of 0.1962 cm^2^ modified by the catalyst served as the working electrode [47, 60].

In addition, a rotating ring‐disk electrode (RRDE) with the same disk area was used to determine the H_2_O_2_ yield and electron transfer number, where the catalyst ink was drop‐cast only onto the central disk region [48, 61].

To prepare the working electrodes, 5 mg of the as‐prepared catalyst and 20 µL of 5 wt.% Nafion solution was dispersed in 980 µL of an isopropanol/water mixture (v/v = 1:2) and ultrasonicated for 3 h to form a homogeneous catalyst ink. Then, 10 µL of the ink was drop‐cast onto the glassy carbon disk of the RDE in several aliquots and dried at room temperature to obtain the catalyst‐coated working electrode [28, 62, 63]. To ensure a fair comparison, all benchmark catalysts (commercial 20 wt.% Pt/C, commercial RuO_2_, and the mixed Pt/C + RuO_2_ catalyst) were fabricated using the same protocol for the as‐prepared catalysts.

The RRDE electrodes were prepared in the same manner, except that the ink was confined to the disk area.

The ORR activity was examined in O_2_‐saturated 0.1 M KOH solution by cyclic voltammetry (CV) and linear sweep voltammetry (LSV) at a scan rate of 5 mV s^−1^. All measured potentials were converted to the reversible hydrogen electrode (RHE) scale using Equation (1) [64]:

(1) $$E_{\mathrm{RHE}}=E_{\mathrm{Hg}/\mathrm{HgO}}+0.098+0.0591\;\mathrm{pH}$$

The ORR durability of FeGa/C‐0.75 and commercial 20 wt.% Pt/C was compared by chronoamperometric measurements at 0.6 V vs. RHE and a rotation rate of 1600 rpm [65].

Tafel plots for ORR were derived from the ORR LSV polarization curves. ORR LSV curves were recorded in O_2_‐saturated 0.1 M KOH at a scan rate of 5 mV s^−1^ and a rotation rate of 1600 rpm, and the potentials were converted to the RHE scale. Tafel plots were constructed by plotting the potential E (or overpotential *η*) versus log *j*, and the Tafel slope was obtained from the linear region at low overpotentials [66].

The OER activity was evaluated in N_2_‐saturated 1 M KOH solution by LSV at a scan rate of 5 mV s^−1^ and a rotation rate of 1600 rpm using the same three‐electrode configuration as for ORR. During the OER measurements, iR compensation (95%, based on R_s_ from EIS) was applied to correct for the ohmic drop in the electrolyte [67].

Tafel plots for OER were derived from the iR‐corrected OER polarization curves. Specifically, the overpotential (η = E – 1.23 V, after conversion to the RHE scale) was plotted as a function of log *j* (geometric current density), and the Tafel slope was obtained from the linear region at low overpotentials [68].

The Koutecký–Levich (K‐L) equations (Equations (2) and (3)) were employed to calculate the electron transfer numbers (n).

(2) $$\frac{1}{j}=\frac{1}{j_{k}}+\frac{1}{j_{L}}=\frac{1}{j_{k}}+\frac{1}{B\;\omega^{1/2}}$$

(3) $$B=0.62\;nFD_{O_{2}}^{2/3}\;\nu^{-1/6}\;C_{O_{2}}$$

where *j* is the measured current density, *j_L_* is the diffusion‐limited current density, and *j_k_* ​is the kinetic current density. *ω* is the angular rotation rate of the electrode, *n* is the number of electrons transferred per O_2_ molecule, *F* is the Faraday constant, *D_(O2)_* is the diffusion coefficient of dissolved O_2_, *ν* is the kinematic viscosity of the electrolyte, and *C_(O2)_* is the bulk concentration of dissolved O_2_ [69].

The H_2_O_2_ yield and electron transfer number (*n*) during ORR were determined using a rotating ring‐disk electrode (RRDE) configuration in O_2_‐saturated 0.1 M KOH. ORR polarization curves were recorded at a scan rate of 5 mV s^−1^ and a rotation rate of 1600 rpm. The disk electrode was coated with the catalyst ink in the same way as for the RDE measurements, while the Pt ring was held at 1.30 V (vs. RHE) to quantitatively oxidize the H_2_O_2_ generated at the disk. The H_2_O_2_ yield and *n* were calculated from the disk current (*I_D_*) and ring current (*I_R_*) according to the following equations:

(4) $$H_{2}O_{2}\;\left(\%\right)=\frac{200I_{R}}{N\;I_{D}+I_{R}}\;$$

(5) $$n=\frac{4NI_{D}}{NI_{D}+I_{R}}\;$$

where *N* is the collection efficiency of the RRDE (0.37 for the RRDE used in this work), *I_D_* is the disk current, and *I_R_* is the ring current. In these equations, a higher *n* value (approaching 4) and a lower H_2_O_2_ yield indicate a more favorable four‐electron ORR pathway [70].

EIS measurements were carried out on a CHI 760E electrochemical workstation using the same three‐electrode configuration as in the OER tests. The catalyst‐coated glassy carbon electrode was used as the working electrode, a Hg/HgO electrode as the reference electrode, and a graphite rod as the counter electrode. The impedance spectra were recorded in N_2_‐saturated 1.0 M KOH at a fixed potential of 1.60 V (vs. RHE), which lies in the OER‐relevant potential region. A small AC perturbation of 5 mV was applied over a frequency range from 100 kHz to 0.1 Hz [71].

The electrochemical double‐layer capacitance was estimated from cyclic voltammograms recorded in a non‐faradaic potential window (1.00–1.10 V vs. RHE) in N2‐saturated electrolyte. CVs were collected at scan rates of 40, 60, 80, 100, and 120 mV s^−1^ [72].

The obtained Nyquist plots were fitted using a simplified Randles equivalent circuit, *R_s_*‐(*R_ct_* ∥*CPE*), where *R_s_* represents the solution resistance, *R_ct_* is the charge‐transfer resistance associated with the interfacial OER process, and *CPE* is a constant phase element describing the non‐ideal double‐layer capacitance and surface heterogeneity. The fitting was performed using ZView software (Scribner Associates, Inc.), and the R*_ct_* values extracted from the fitted spectra were used to compare the charge‐transfer kinetics of the different catalysts.

### 4.4 First‐Principles Calculations

All density functional theory (DFT) calculations were performed using the Vienna Ab initio Simulation Package (VASP) [73]. The electron‐ion interaction was described using the projector augmented‐wave (PAW) method [74]. Exchange‐correlation effects were treated with the Perdew–Burke–Ernzerhof (PBE) functional, and dispersion interactions were included using the DFT‐D3 correction [75, 76, 77]. A plane‐wave cutoff energy of 520 eV was used. Brillouin‐zone sampling employed a Monkhorst‐Pack k‐point mesh of 2 × 2 × 1 [78]. Based on the XRD structure, we constructed a multilayer slab model consisting of Fe_4_N(110), GaN(100), and bilayer graphene. All structures were fully relaxed until the residual forces on each atom were below 0.02 eV Å^−1^. Spin polarization and dipole corrections were included in all calculations. A vacuum spacing of >15 Å was applied along the surface‐normal direction to avoid spurious interactions between periodic images. The optimized in‐plane lattice parameters of the supercell were a = 8.871373 Å and b = 10.019925 Å, as summarized in Table S6. Gibbs free‐energy profiles were evaluated following the approach pioneered by Nørskov and co‐workers by adding standard entropic contributions of gas‐phase species to the calculated DFT energies [79, 80, 81, 82, 83, 84, 85, 86, 87]. Given the structural complexity of experimental catalysts, the calculated model represents a stable, catalytically relevant motif that captures the qualitative trends of the observed bifunctional behavior.

### 4.5 Aqueous Rechargeable Zinc‐Air Battery Assembly and Measurements

Aqueous rechargeable ZABs were assembled using FeGa/C‐0.75 or a mixed Pt/C + RuO_2_ catalyst (mass ratio 1:1) as the air cathode and a polished Zn plate as the anode. The air cathodes were prepared by drop‐casting the catalyst ink onto carbon paper to obtain a catalyst loading of 1.0 mg cm^−2^ (based on the total catalyst mass) and drying at room temperature. This carbon paper was laminated with a waterproof and breathable membrane, and a piece of Ni foam was attached to the outermost side as the current collector/lead for electrical connection. The Zn plate was mechanically polished with sandpaper and rinsed with deionized water prior to use. A 6.0 M KOH + 0.2 M Zn(CH_3_COO)_2_ aqueous solution was used as the electrolyte. The Zn anode, electrolyte, and catalyst‐coated carbon paper were assembled into a commercial polymethyl methacrylate (PMMA) ZAB fixture, with the catalyst layer facing the electrolyte and the gas side of the air electrode exposed to ambient air [88]. Unless otherwise specified, the aqueous ZABs were tested at 25°C with an effective air‐cathode area of 1.0 cm^2^, 3.0 mL electrolyte, a Zn plate thickness of 1.0 mm, and a Ni foam current collector thickness of 0.5 mm.

The electrochemical performance of the assembled ZABs was first evaluated on a CHI 760E electrochemical workstation in a two‐electrode configuration, with the Zn plate as the anode and the air cathode (FeGa/C‐0.75 or Pt/C + RuO_2_) as the cathode. The open‐circuit voltage (OCV) was recorded after the cell voltage had stabilized. Discharge polarization and the corresponding power density curves were obtained by linear sweep voltammetry (LSV) at a scan rate of 5 mV s^−1^. The power density (*P*) was calculated from *P* = *I* × *V* / *A*, where *I* is the discharge current, *V* is the cell voltage, and *A* is the geometric area of the air cathode. Short‐term galvanostatic charge–discharge profiles were also recorded on the CHI 760E to compare the round‐trip efficiency of ZABs with FeGa/C‐0.75 and Pt/C + RuO_2_ air cathodes [89].

Long‐term charge–discharge cycling and specific capacity measurements were carried out using a Land‐2001A battery testing system (Wuhan, China). For the specific capacity tests, the ZABs were discharged galvanostatically at 20 mA cm^−2^ until a cut‐off voltage of 0.6 V, and the specific capacity was calculated based on the consumed Zn mass (*m_Zn_*) according to *C* (mAh g_Zn_^−1^) = *I* × *t* / *m_Zn_*, where *I* is the discharge current and *t* is the discharge time. The long‐term cycling performance of the rechargeable ZABs was evaluated by repeated galvanostatic charge–discharge at a current density of 30 mA cm^−2^, with each charge and discharge step lasting 1 h [90].

### 4.6 Flexible All‐Solid‐State Zinc‐Air Battery Assembly

Flexible ZABs were assembled using FeGa/C‐0.75 as the air cathode, a mechanically polished thin Zn foil as the flexible anode, and a PAA‐KOH gel as the solid electrolyte. The flexible air cathodes were prepared by drop‐casting the catalyst ink onto flexible carbon paper and drying at room temperature, giving a catalyst loading identical to that used for the aqueous ZABs (1.0 mg cm^−2^).

The PAA‐KOH gel electrolyte was obtained by allowing a pre‐formed PAA film to fully absorb 6 M KOH solution until a homogeneous, transparent gel was formed. For cell assembly, the Zn foil, PAA‐KOH gel electrolyte, and FeGa/C‐0.75‐coated flexible carbon paper were stacked in sequence to form a sandwich structure, with the catalyst layer facing the gel electrolyte. The entire cell was then encapsulated using a heat‐sealing plastic film, in which small gas‐permeable openings were reserved on the air side to ensure sufficient exposure of the air electrode to ambient oxygen, thus yielding a bendable solid‐state flexible ZAB. For demonstration, a single flexible ZAB was also used to continuously power a commercial red LED under repeated bending and straightening conditions.

## Author Contributions

**Xin‐Yuan Wei**: conceptualization, methodology, investigation, Writing – review and editing, Writing – original draft. **Sai‐Sai Xie**: investigation, writing – review and editing. **Xia‐Li Ding**: supervision, resources. **Yuan‐Qi Zhai**: writing – review and editing, supervision, formal analysis. **Sen Yu**: resources, supervision. **Xiang‐Quan Hu**: formal analysis. **Rong‐Qian Wu**: investigation. **Jintao Lu**: formal analysis. **Yan‐Zhen Zheng**: writing – review and editing, funding acquisition, supervision, resources.

## Conflicts of Interest

The authors declare no conflicts of interest.

## Supporting information




**Supporting File 1**: advs76564‐sup‐0001‐SuppMat.docx.

**Supporting File 2**: advs76564‐sup‐0002‐MovieS1.mp4.

**Supporting File 3**: advs76564‐sup‐0003‐MovieS2.mp4.

## Data Availability

The data that support the findings of this study are available from the corresponding author upon reasonable request.
