# Supplementary material for: (Fe4N/GaN)@GC: A Bifunctional Electrocatalyst for High‐Performance Rechargeable Zinc‐Air Batteries
Source: Adv Sci (Weinh). 2026 Jul 16;13(55):e76564. doi: 10.1002/advs.76564 (PMC13373900; doi:10.1002/advs.76564)
Supplement: Supplementary file 1 [file ADVS-13-e76564-s003.docx]

**(Fe_4_N/GaN)@GC: A Bifunctional Electrocatalyst for High-Performance Rechargeable Zinc-Air Batteries**

*Xin-Yuan Wei, Sai-Sai Xie, Xia-Li Ding*, Yuan-Qi Zhai*, Sen Yu, Xiang-Quan Hu, Rong-Qian Wu, Jintao Lu and Yan-Zhen Zheng**

**Fig. S1. Phase evolution across the FeGa/C-x series as a function of Ga feed ratio.** XRD patterns of FeGa/C-x samples with different stoichiometric ratios (x = 0.25, 0.5, 0.75, 1.0).

**Fig. S2. Crystallographic evidence for the coexistence of Fe_4_N and GaN in FeGa/C-0.75.** Selected-area electron diffraction (SAED) pattern of FeGa/C-0.75. The diffraction rings can be indexed to cubic Fe_4_N and wurtzite GaN phases, indicating the coexistence of Fe_4_N and GaN in the composite. The ring assignments are consistent with the lattice-fringe observations in the HRTEM images (Fig. 1f).

**Table S1. Indexing of SAED diffraction rings for FeGa/C-0.75.** The selected-area electron diffraction (SAED) pattern shows multiple diffraction rings that can be indexed to cubic Fe_4_N and wurtzite GaN phases. The measured scattering vectors (q), calculated interplanar spacings (d), and corresponding lattice planes are summarized below.

| Ring | q(nm^-1^) | d(nm) | Assigned phase | hkl |
| --- | --- | --- | --- | --- |
| 1 | 2.73 | 0.366 | Fe_4_N | 100 |
| 2 | 3.54 | 0.283 | GaN | 100 |
| 3 | 3.78 | 0.264 | GaN | 002 |
| 4 | 3.96 | 0.253 | GaN | 101 |
| 5 | 4.41 | 0.227 | Fe_4_N | 111 |
| 6 | 6.11 | 0.164 | Fe_4_N | 210 |
| 7 | 6.71 | 0.149 | GaN | 103 |
| 8 | 7.25 | 0.138 | GaN | 200 |

**Fig. S3. Raman signatures of carbon structure and defect density in control and heterostructured catalysts.** Raman spectroscopy of C, Ga/C, Fe/C, and FeGa/C-0.75.

**Fig. S4. Representative morphology of FeGa/C-0.75 catalyst clusters.** (A) TEM image of the FeGa/C-0.75 catalyst cluster. (B) Enlarged TEM image of the same FeGa/C-0.75 cluster.

**Fig. S5. XPS survey spectra confirming the elemental composition of control and heterostructured catalysts.** XPS survey of C, Ga/C, Fe/C, and FeGa/C-0.75.

**Fig. S6. Redox features of FeGa/C-0.75 under alkaline electrochemical conditions.** Cyclic voltammetry (CV) measurements of FeGa/C-0.75.

**Fig. S7. Composition-dependent ORR activity in the FeGa/C-x series.** ORR activity of FeGa/C-x (x = 0, 0.25, 0.5, 0.75, 1.0), x = 0 represents the Fe/C sample.

**Fig. S8. ORR kinetics of FeGa/C-x catalysts quantified by Tafel analysis.** ORR Tafel of FeGa/C-x (x = 0, 0.25, 0.5, 0.75, 1.0).

**Fig. S9. Composition-dependent OER activity in the FeGa/C-x series.** OER activity of FeGa/C-x (x = 0, 0.25, 0.5, 0.75, 1.0).

**Fig. S10. OER kinetics of FeGa/C-x catalysts quantified by Tafel analysis.** OER Tafel of FeGa/C-x (x = 0, 0.25, 0.5, 0.75, 1.0).

**Fig. S11. Interfacial charge-transfer characteristics of control and heterostructured catalysts probed by EIS.** Electrochemical impedance spectroscopy (EIS) measurements of C, Ga/C, Fe/C, and FeGa/C-0.75 at 1.6 V vs. RHE under 1 M KOH. Nyquist plots were fitted using a simplified Randles equivalent circuit (Rs-(Rct//CPE)).

**Fig. S12. Zn anode morphology before and after the specific-capacity test.**

**Fig. S13. Open-circuit voltage of the FeGa/C-0.75-based flexible ZAB.**

**Fig. S14. Double-layer capacitance measurements used to estimate ECSA of FeGa/C-x catalysts.** Scan-rate-dependent cyclic voltammograms recorded in the non-faradaic region (1.00 - 1.10 V vs. RHE) in N_2_-saturated electrolyte for (A) Fe/C, (B) FeGa/C-0.25, (C) FeGa/C-0.5, (D) FeGa/C-0.75, and (E) FeGa/C-1.0 at scan rates of 40-120 mV s^-1^. (F) ECSA-normalized ORR polarization curves of FeGa/C-x catalysts. (G) ECSA-normalized OER polarization curves of FeGa/C-x catalysts. The ECSA-normalized current density was calculated by dividing the geometric current density by the Cdl-derived ECSA.

**Fig. S15. Phase purity of the HMTA-assisted Fe_4_N control catalyst verified by XRD.** XRD patterns of Fe/C-HMTA.

**Fig. S16. Effect of non–in situ contact with GaN on ORR and OER activity of the Fe_4_N control.** **(A)** ORR polarization curves of Fe/C-HMTA and Fe/C-HMTA+GaN in O_2_-saturated 0.1 M KOH at 1600 rpm. **(B)** OER LSV curves after iR compensation of Fe/C-HMTA and Fe/C-HMTA+GaN in 1.0 M KOH (rotation rate: 1600 rpm; scan rate: 5 mV s^-1^).

**Fig. S17.** **Structural and compositional characterization of FeGa/C-0.75 after OER operation.** (a,b) HRTEM images of the catalyst after the OER test. The carbon-encapsulated nanoparticle morphology is largely maintained, and crystalline lattice fringes can still be observed, although slight local amorphization is present. (c) Superimposed EDS elemental mapping image. (d–f) EDS elemental maps of N, Fe, and Ga, respectively.

Fe and Ga remain spatially co-localized in the post-OER sample without obvious elemental segregation or severe metal leaching, indicating that the Fe_4_N/GaN-derived heterostructure remains largely preserved after OER operation.

**Fig. S18. LSV curves of FeGa/C-0.75 at various rotation rates in O_2_-saturated 0.1 M KOH; inset: corresponding Koutecky-Levich plots at different potentials.**

**Fig. S19. ORR LSV curves of FeGa/C-0.75 at the 1st and 3000th cycles.**

**Fig. S20. Operando Raman spectra of FeGa/C-0.75 under ORR and OER conditions.** (a) Operando Raman spectra collected under ORR-relevant potentials from OCP to 0.5 V vs. RHE. (b) Operando Raman spectra collected under OER-relevant potentials from OCP to 1.7 V vs. RHE. The spectra were baseline-corrected and vertically shifted for clarity. The shaded regions indicate low-wavenumber oxygenated adsorbate-related vibrations. During ORR polarization, reversible M-O(H)-related features are observed, suggesting the participation of oxygenated adsorbates during oxygen reduction. During OER polarization, weak and broad M-O(H)-related/oxygenated-species signals become detectable mainly at high anodic potentials, indicating partial surface oxygenation or adsorbed oxygenated species under OER operation. The persistent D and G bands indicate that the carbon framework remains spectroscopically detectable during electrochemical polarization.

**Fig. S21. Low-magnification TEM-EDS elemental mapping of FeGa/C-0.75.** Low-magnification TEM image and corresponding EDS elemental maps of N, Fe, and Ga for FeGa/C-0.75. The Fe, Ga, and N elements are broadly co-distributed over the catalyst aggregate, indicating the successful incorporation of Fe- and Ga-containing nitride species at the mesoscale.

**Fig. S22. Aberration-corrected STEM characterizations of FeGa/C-0.75.** (a,b) Aberration-corrected ADF-STEM images and corresponding STEM-EDS elemental maps of N, Fe, and Ga collected from representative local regions of FeGa/C-0.75. The lattice spacings of 0.218–0.219 nm and 0.242-0.243 nm are assigned to Fe_4_N (111) and GaN (101), respectively. The adjacent Fe- and Ga-containing crystalline domains with coexisting N signals provide additional evidence for the local coupling of Fe_4_N and GaN and exclude severe particle-level elemental segregation. (c,d) Representative high-resolution ADF-STEM images of carbon-encapsulated Fe_4_N nanodomains. The crystalline Fe_4_N domains are wrapped by thin carbon shells, and no obvious isolated metal-related atomic contrast is observed on the inspected carbon-shell regions, suggesting that the dominant Fe species are present as crystalline nitride nanodomains without obvious atomically dispersed Fe species decorating the carbon surface.

**Table S2. The element contents of C, Ga/C, Fe/C and FeGa/C-0.75 catalysts from XPS results.**

| Catalysts | Total C  (at.%) | Total N  (at.%) | Total Ga  (at.%) | Total Fe  (at.%) |
| --- | --- | --- | --- | --- |
| C | 52.12 | 47.87 | - | - |
| Ga/C | 25.3 | 39.73 | 34.98 | - |
| Fe/C | 84.7 | 5.56 | - | 9.75 |
| FeGa/C-0.75 | 53.35 | 22.17 | 20.72 | 3.77 |

**Table S3. Deconvoluted N 1s peak area fractions of C, Ga/C, Fe/C and FeGa/C-0.75 catalysts calculated from XPS analysis.**

| N species  percentage(%) | C | Ga/C | Fe/C | FeGa/C-0.75 |
| --- | --- | --- | --- | --- |
| Graphitic N | 14.07 | - | 29.32 | 3.74 |
| Pyridinic N | 59.79 | 34.73 | 32.19 | 37.8 |
| Pyrrolic N | 18.76 | 16.44 | 11.69 | 3.25 |
| Oxidized N | 7.37 | - | 12.29 | 1.4 |
| Ga-N | 0 | 34.68 | - | 48.4 |
| Metal-N | 0 | 14.15 | 14.21 | 5.41 |

**Table S4. ICP-OES analysis of Fe and Ga contents in FeGa/C-x series.**

| **Table S4** ICP-OES analysis of Fe and Ga contents in FeGa/C-x series. | | | |
| --- | --- | --- | --- |
| Catalysts | Fe (%) | Ga (%) | Ga/Fe (molar ratio) |
| FeGa/C-0 | 68.6 | - |  |
| FeGa/C-0.25 | 55.42 | 13.88 | 0.2 |
| FeGa/C-0.5 | 47.78 | 22.17 | 0.37 |
| FeGa/C-0.75 | 40.57 | 27.85 | 0.55 |
| FeGa/C-1 | 35.51 | 33.93 | 0.77 |

**Table S5. The ECSA values estimated from the double-layer capacitance (Cdl) of FeGa/C-x series.**

| **Table S5** The ECSA values of FeGa/C-x series. | | | |
| --- | --- | --- | --- |
| Catalysts | Cdl (mF cm⁻²) | R² | Cdl-derived relative ECSA (cm²) |
| Fe/C | 3.00 | 0.9998 | 74.99 |
| FeGa/C-0.25 | 1.65 | 0.9982 | 41.15 |
| FeGa/C-0.5 | 2.47 | 0.9975 | 61.63 |
| FeGa/C-0.75 | 1.87 | 0.9943 | 46.82 |
| FeGa/C-1.0 | 1.71 | 0.998 | 42.78 |

**Table S6. Structural parameters of the DFT models used in the calculations.**

| **Table S6** Structural parameters of the DFT models. | | | | | |
| --- | --- | --- | --- | --- | --- |
|  |  |  |  |  |  |
| **Model** | **Surface/slab** | **Cell parameters** | **Atom number** | **Vacuum** | **Use** |
| Fe_4_N/C reference model | Fe_4_N(110) + bilayer graphene | a = 8.871373 Å, b = 10.019925 Å, c = 32.000000 Å | Fe 48, N 12, C 32 | >15 Å | Reference model |
| Fe_4_N/GaN/C model | Fe_4_N(110) + GaN(100) + bilayer graphene | a = 8.871373 Å, b = 10.019925 Å, c = 32.000000 Å | Fe 48, Ga 24, N 36, C 32 | >15 Å | GaN-modified model |
| ORR-*OH model | *OH adsorbed on interfacial Fe-N site | Same as Fe_4_N/GaN/C | Fe 48, Ga 24, N 36, C 32, O 1, H 1 | >15 Å | ORR intermediate |
| ORR-*O model | *O adsorbed on interfacial Fe-N site | Same as Fe_4_N/GaN/C | Fe 48, Ga 24, N 36, C 32, O 1 | >15 Å | ORR intermediate |
| ORR-*OOH model | *OOH adsorbed on interfacial Fe-N site | Same as Fe_4_N/GaN/C | Fe 48, Ga 24, N 36, C 32, O 2, H 1 | >15 Å | ORR intermediate |
| OER-*OH model | *OH adsorbed on carbon-shell/interfacial C site | Same as Fe_4_N/GaN/C | Fe 48, Ga 24, N 36, C 32, O 1, H 1 | >15 Å | OER intermediate |
| OER-*O model | *O adsorbed on carbon-shell/interfacial C site | Same as Fe_4_N/GaN/C | Fe 48, Ga 24, N 36, C 32, O 1 | >15 Å | OER intermediate |
| OER-*OOH model | *OOH adsorbed on carbon-shell/interfacial C site | Same as Fe_4_N/GaN/C | Fe 48, Ga 24, N 36, C 32, O 2, H 1 | >15 Å | OER intermediate |
| A vacuum region larger than 15 Å was introduced along the surface-normal direction for all slab models to reduce artificial periodic interactions. For all models listed above, α = β = 90.000°, and γ ≈ 90.000°. | | | | | |

**Table S7 Atomic coordinates of the Fe_4_N/GaN/C model used in the calculations.**

| No | Atom | Element | x_fractional | y_fractional | z_fractional |
| --- | --- | --- | --- | --- | --- |
| 1 | Fe1 | Fe | 0.16674 | 0.393449 | 0.437529 |
| 2 | Fe2 | Fe | 0.166613 | 0.190608 | 0.557768 |
| 3 | Fe3 | Fe | 0.000461 | 0.060736 | 0.514371 |
| 4 | Fe4 | Fe | -0.00024 | 0.310937 | 0.609528 |
| 5 | Fe5 | Fe | 0.166935 | 0.139256 | 0.460355 |
| 6 | Fe6 | Fe | 0.166568 | 0.424235 | 0.55661 |
| 7 | Fe7 | Fe | -5.3E-05 | 0.305166 | 0.497616 |
| 8 | Fe8 | Fe | 0.000297 | 0.060307 | 0.606599 |
| 9 | Fe9 | Fe | 0.498254 | 0.392491 | 0.437569 |
| 10 | Fe10 | Fe | 0.499846 | 0.191218 | 0.557611 |
| 11 | Fe11 | Fe | 0.332774 | 0.060483 | 0.514471 |
| 12 | Fe12 | Fe | 0.333346 | 0.311062 | 0.609644 |
| 13 | Fe13 | Fe | 0.499025 | 0.139454 | 0.460594 |
| 14 | Fe14 | Fe | 0.500217 | 0.425524 | 0.556216 |
| 15 | Fe15 | Fe | 0.332989 | 0.305037 | 0.497593 |
| 16 | Fe16 | Fe | 0.332806 | 0.060299 | 0.606682 |
| 17 | Fe17 | Fe | 0.834876 | 0.392255 | 0.437588 |
| 18 | Fe18 | Fe | 0.83318 | 0.191202 | 0.55755 |
| 19 | Fe19 | Fe | 0.666378 | 0.060951 | 0.514096 |
| 20 | Fe20 | Fe | 0.666509 | 0.312235 | 0.609247 |
| 21 | Fe21 | Fe | 0.833689 | 0.139619 | 0.460593 |
| 22 | Fe22 | Fe | 0.832914 | 0.425488 | 0.556152 |
| 23 | Fe23 | Fe | 0.666532 | 0.305611 | 0.497247 |
| 24 | Fe24 | Fe | 0.666561 | 0.061151 | 0.606383 |
| 25 | Fe25 | Fe | 0.166845 | 0.893582 | 0.437372 |
| 26 | Fe26 | Fe | 0.166573 | 0.690715 | 0.557024 |
| 27 | Fe27 | Fe | 0.000833 | 0.559537 | 0.514158 |
| 28 | Fe28 | Fe | -0.00025 | 0.810366 | 0.609073 |
| 29 | Fe29 | Fe | 0.166982 | 0.639872 | 0.460415 |
| 30 | Fe30 | Fe | 0.166481 | 0.92517 | 0.556546 |
| 31 | Fe31 | Fe | 1.04E-05 | 0.806423 | 0.497307 |
| 32 | Fe32 | Fe | 0.000461 | 0.561027 | 0.606135 |
| 33 | Fe33 | Fe | 0.49773 | 0.893273 | 0.437376 |
| 34 | Fe34 | Fe | 0.499916 | 0.691032 | 0.556813 |
| 35 | Fe35 | Fe | 0.332337 | 0.559203 | 0.514225 |
| 36 | Fe36 | Fe | 0.333197 | 0.810548 | 0.609172 |
| 37 | Fe37 | Fe | 0.496352 | 0.639959 | 0.460535 |
| 38 | Fe38 | Fe | 0.500203 | 0.925673 | 0.556278 |
| 39 | Fe39 | Fe | 0.332933 | 0.806396 | 0.497306 |
| 40 | Fe40 | Fe | 0.332729 | 0.560951 | 0.60625 |
| 41 | Fe41 | Fe | 0.835149 | 0.893088 | 0.437416 |
| 42 | Fe42 | Fe | 0.833059 | 0.690918 | 0.556787 |
| 43 | Fe43 | Fe | 0.666354 | 0.560496 | 0.513417 |
| 44 | Fe44 | Fe | 0.666535 | 0.811769 | 0.608963 |
| 45 | Fe45 | Fe | 0.836148 | 0.64006 | 0.460543 |
| 46 | Fe46 | Fe | 0.833014 | 0.925669 | 0.556194 |
| 47 | Fe47 | Fe | 0.666545 | 0.806502 | 0.49661 |
| 48 | Fe48 | Fe | 0.666583 | 0.562015 | 0.605953 |
| 49 | N1 | N | 0.166618 | 0.952127 | 0.493769 |
| 50 | N2 | N | 0.166532 | 0.436316 | 0.616323 |
| 51 | N3 | N | 0.499666 | 0.953031 | 0.493503 |
| 52 | N4 | N | 0.499818 | 0.437218 | 0.616012 |
| 53 | N5 | N | 0.833369 | 0.953151 | 0.493513 |
| 54 | N6 | N | 0.8333 | 0.437367 | 0.615893 |
| 55 | N7 | N | 0.166465 | 0.450056 | 0.493981 |
| 56 | N8 | N | 0.166489 | 0.93567 | 0.616232 |
| 57 | N9 | N | 0.499547 | 0.451365 | 0.493659 |
| 58 | N10 | N | 0.499697 | 0.936395 | 0.61602 |
| 59 | N11 | N | 0.833544 | 0.451597 | 0.493634 |
| 60 | N12 | N | 0.833302 | 0.9366 | 0.615909 |
| 61 | N13 | N | 4.77E-05 | 0.369861 | 0.291244 |
| 62 | N14 | N | 0.166677 | 0.367644 | 0.379935 |
| 63 | N15 | N | 0.166829 | 0.127588 | 0.256192 |
| 64 | N16 | N | 7.1E-05 | 0.122082 | 0.348224 |
| 65 | N17 | N | 0.333357 | 0.369925 | 0.291286 |
| 66 | N18 | N | 0.499718 | 0.367473 | 0.379927 |
| 67 | N19 | N | 0.500175 | 0.127717 | 0.256321 |
| 68 | N20 | N | 0.333381 | 0.122154 | 0.348227 |
| 69 | N21 | N | 0.666718 | 0.370003 | 0.291515 |
| 70 | N22 | N | 0.833691 | 0.367434 | 0.379926 |
| 71 | N23 | N | 0.833174 | 0.127647 | 0.256347 |
| 72 | N24 | N | 0.666696 | 0.122132 | 0.348442 |
| 73 | N25 | N | 6.54E-05 | 0.869983 | 0.291159 |
| 74 | N26 | N | 0.166683 | 0.86774 | 0.379802 |
| 75 | N27 | N | 0.166829 | 0.627395 | 0.2562 |
| 76 | N28 | N | 8.93E-05 | 0.622156 | 0.348217 |
| 77 | N29 | N | 0.333364 | 0.870067 | 0.291193 |
| 78 | N30 | N | 0.499785 | 0.867627 | 0.379799 |
| 79 | N31 | N | 0.500239 | 0.627598 | 0.256376 |
| 80 | N32 | N | 0.333334 | 0.622198 | 0.348214 |
| 81 | N33 | N | 0.666758 | 0.87013 | 0.291443 |
| 82 | N34 | N | 0.833645 | 0.867704 | 0.379803 |
| 83 | N35 | N | 0.83312 | 0.627535 | 0.256391 |
| 84 | N36 | N | 0.666743 | 0.622149 | 0.348501 |
| 85 | Ga1 | Ga | 0.000132 | 0.180669 | 0.288655 |
| 86 | Ga2 | Ga | 0.166684 | 0.172929 | 0.383488 |
| 87 | Ga3 | Ga | 0.16673 | 0.449519 | 0.26593 |
| 88 | Ga4 | Ga | 8.2E-05 | 0.432822 | 0.349873 |
| 89 | Ga5 | Ga | 0.333371 | 0.18073 | 0.288711 |
| 90 | Ga6 | Ga | 0.499806 | 0.172899 | 0.383589 |
| 91 | Ga7 | Ga | 0.500205 | 0.449688 | 0.266115 |
| 92 | Ga8 | Ga | 0.33328 | 0.432905 | 0.349907 |
| 93 | Ga9 | Ga | 0.666669 | 0.180825 | 0.288857 |
| 94 | Ga10 | Ga | 0.833641 | 0.17282 | 0.383595 |
| 95 | Ga11 | Ga | 0.833181 | 0.449532 | 0.266061 |
| 96 | Ga12 | Ga | 0.666719 | 0.432742 | 0.350157 |
| 97 | Ga13 | Ga | 0.000146 | 0.68083 | 0.288598 |
| 98 | Ga14 | Ga | 0.166686 | 0.673133 | 0.383472 |
| 99 | Ga15 | Ga | 0.166726 | 0.949679 | 0.26582 |
| 100 | Ga16 | Ga | 0.000115 | 0.932798 | 0.349758 |
| 101 | Ga17 | Ga | 0.33335 | 0.680905 | 0.288653 |
| 102 | Ga18 | Ga | 0.499731 | 0.673181 | 0.383562 |
| 103 | Ga19 | Ga | 0.500249 | 0.949801 | 0.266018 |
| 104 | Ga20 | Ga | 0.33329 | 0.932904 | 0.349801 |
| 105 | Ga21 | Ga | 0.666685 | 0.680969 | 0.28887 |
| 106 | Ga22 | Ga | 0.83374 | 0.673237 | 0.383581 |
| 107 | Ga23 | Ga | 0.83321 | 0.949631 | 0.265959 |
| 108 | Ga24 | Ga | 0.666708 | 0.932786 | 0.350022 |
| 109 | C1 | C | 0.000775 | 0.000252 | 0.712331 |
| 110 | C2 | C | 0.251022 | 0.125106 | 0.711893 |
| 111 | C3 | C | 0.332889 | 0.000234 | 0.712313 |
| 112 | C4 | C | 0.082643 | 0.12512 | 0.711902 |
| 113 | C5 | C | 0.500947 | 0.000209 | 0.712966 |
| 114 | C6 | C | 0.750999 | 0.125096 | 0.712949 |
| 115 | C7 | C | 0.832726 | 0.000193 | 0.712976 |
| 116 | C8 | C | 0.582674 | 0.125107 | 0.712946 |
| 117 | C9 | C | 0.000959 | 0.250237 | 0.712012 |
| 118 | C10 | C | 0.250931 | 0.375292 | 0.712066 |
| 119 | C11 | C | 0.332724 | 0.250236 | 0.712001 |
| 120 | C12 | C | 0.082738 | 0.375259 | 0.712072 |
| 121 | C13 | C | 0.501199 | 0.250238 | 0.712775 |
| 122 | C14 | C | 0.750947 | 0.375193 | 0.713203 |
| 123 | C15 | C | 0.832476 | 0.250229 | 0.712779 |
| 124 | C16 | C | 0.58273 | 0.375161 | 0.713205 |
| 125 | C17 | C | 0.000775 | 0.500241 | 0.712342 |
| 126 | C18 | C | 0.251018 | 0.625079 | 0.711933 |
| 127 | C19 | C | 0.332882 | 0.500234 | 0.712339 |
| 128 | C20 | C | 0.082654 | 0.625105 | 0.711938 |
| 129 | C21 | C | 0.500971 | 0.500199 | 0.712978 |
| 130 | C22 | C | 0.750991 | 0.625085 | 0.712937 |
| 131 | C23 | C | 0.832697 | 0.500195 | 0.712975 |
| 132 | C24 | C | 0.582689 | 0.625107 | 0.71294 |
| 133 | C25 | C | 0.000935 | 0.750216 | 0.712046 |
| 134 | C26 | C | 0.250878 | 0.875286 | 0.712082 |
| 135 | C27 | C | 0.332749 | 0.750217 | 0.712034 |
| 136 | C28 | C | 0.082791 | 0.875248 | 0.712095 |
| 137 | C29 | C | 0.501149 | 0.750226 | 0.712772 |
| 138 | C30 | C | 0.750912 | 0.875198 | 0.713198 |
| 139 | C31 | C | 0.832527 | 0.750221 | 0.712774 |
| 140 | C32 | C | 0.582764 | 0.875163 | 0.713195 |

**Movie S1. Demonstration of the flexible battery powering an LED.**

**Movie S2. Demonstration of the flexible battery attached to body joints.**
